# Yin Yang 1 promotes the neuroendocrine differentiation of prostate cancer cells via the non‐canonical WNT pathway (FYN/STAT3)

**DOI:** 10.1002/ctm2.1422

**Published:** 2023-09-28

**Authors:** Rui‐ji Liu, Zhi‐Peng Xu, Xiang Huang, Bin Xu, Ming Chen

**Affiliations:** ^1^ Department of Urology, Sichuan Provincial People's Hospital, School of Medicine University of Electronic Science and Technology of China Chengdu China; ^2^ Department of Urology Affiliated Zhongda Hospital of Southeast University Nanjing China; ^3^ Surgical Research Center, Institute of Urology Southeast University Medical School Nanjing China; ^4^ Department of Urology, Nanjing Lishui District People's Hospital Zhongda Hospital Lishui Branch Southeast University Nanjing China

**Keywords:** cellular lineage plasticity, EMT, FZD8, neuroendocrine differentiation of prostate cancer, YY1

## Abstract

**Background:**

A growing number of studies have shown that Yin Yang 1 (YY1) promotes the development of multiple tumours. The purpose of the current study was to determine the mechanism by which YY1 mediates neuroendocrine differentiation of prostate cancer (NEPC) cells undergoing cellular plasticity.

**Methods:**

Using the Cancer Genome Atlas and Gene Expression Omnibus (GEO) databases, we bioinformatically analyzed YY1 expression in prostate cancer (PCa). Aberrant YY1 expression was validated in different PCa tissues and cell lines via quantitative reverse transcription polymerase chain reaction, western blotting, and immunohistochemistry. In vivo and in vitro functional assays verified the oncogenicity of YY1 in PCa. Further functional assays showed that ectopic expression of YY1 promoted cellular plasticity in PCa cells via epithelial‐mesenchymal transition induction and neuroendocrine differentiation.

**Results:**

Androgen deprivation therapy induced a decrease in YY1 protein ubiquitination, enhanced its stability, and thus enhanced the transcriptional activity of FZD8. Castration enhanced FZD8 binding to Wnt9A and mediated cellular plasticity by activating the non‐canonical Wnt (FZD8/FYN/STAT3) pathway.

**Conclusions:**

We identified YY1 as a novel dysregulated transcription factor that plays an important role in NEPC progression in this study. We believe that an in‐depth investigation of the mechanism underlying YY1‐mediated disease may lead to improved NEPC therapies.

## Graphical abstract

Androgen deprivation therapy stabilizes Yin Yang 1 (YY1) protein by inhibiting YY1 ubiquitination. In the nucleus, YY1 induces transcription by binding to the promoter‐specific region of FZD8, which activates the non‐canonical Wnt pathway at the cell membrane in association with Wnt9A, allowing phosphorylation of FYN as well as STAT3. Activated STAT3 signaling induces the expression of downstream neuroendocrine markers and epithelial‐mesenchymal transition markers and promotes cell plasticity.

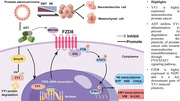

## 1 BACKGROUND

Approximately 1.2 million new cases of prostate cancer (PCa) are diagnosed every year worldwide, resulting in over 350,000 deaths.^1^, ^2^ The increased risk of PCa is positively correlated with age, with more than 85% of patients over 60 years of age at the time of diagnosis.^3^ The androgen‐mediated androgen receptor signalling pathway drives the growth and development of PCa. The establishment of androgen deprivation therapy (ADT) in 1941 by Huggins et al.^4^ created a paradigm shift for PCa treatment that is still used to date. PCa that is initially hormone therapy‐sensitive will ultimately develop into castration‐resistant prostate cancer (CRPC).^4^, ^5^

Histologically, CRPC is primarily classified as an adenocarcinoma. However, a subset of clinical CRPC cases belongs to a histologically variant form of PCa (small‐cell neuroendocrine [NE] carcinoma), which cannot differentiate luminally.^6^ De novo neuroendocrine prostate cancer (NEPC) is uncommon, comprising less than 2% of all diagnosed cases of PCa. The emergence of NE as an ADT‐mediated NE phenotype accounts for 25% of CRPC cases.^7^, ^8^ A hallmark of NEPC is the absence of the androgen receptor (AR) and expression of NE markers, such as synaptophysin (SYP), chromogranin A, neuron‐specific enolase and CD56. NEPC is independent of the AR pathway and evades sensitivity to AR pathway inhibitors.^4^, ^9^ Two prevailing hypotheses offer explanations of the origin of NEPC: NEPC originates from normal NE cells and eventually expands during ADT^10^ and therapy‐driven stress selects genetic and epigenetic dysregulation that drives adenocarcinoma to undergo cell lineage trans‐differentiation to form NE carcinoma.^11^, ^12^ Previous studies have addressed the underlying molecular mechanisms driving cellular plasticity in NE cell trans‐differentiation. Lineage tracing of distinct NE differentiation regions in genetically engineered mouse models with combined Tp53 and Pten deletion have shown NEPC to develop from trans‐differentiating luminal adenocarcinoma cells.^13^ N‐Myc‐induced PCa and NEPC originated from a common prostate epithelial clone.^14^ Indeed, three biological cellular states (epithelial‐mesenchymal transition [EMT], cell stemness and NE nature) are interconnected by a shared network of transcription factors, epigenetic regulators, and cell surface receptors.^13^, ^15^, ^16^

Yin Yang 1 (YY1) is a 414 amino acid‐containing DNA‐binding protein consisting of multiple functional, structural domains and is a member of the GLI‐Krüppel family.^17^, ^18^ YY1 has a dual role in gene regulation, acting as both a transcriptional activator (N‐terminal activation structural domain) and a repressor (C‐terminal repression structural domain).^19^ YY1 is a zinc finger transcription factor that is commonly expressed in normal and many cancerous tissues, but the levels of YY1 are generally higher in cancerous tissues. YY1 is known to regulate approximately 10% of human genes^20^ and has numerous biological functions in tumour development as follows: forms a complex with hypoxia‐inducible factor‐1 and promotes the expression of vascular endothelial growth factor, which regulates tumour angiogenesis^21^; induces p53 ubiquitination and degradation to promote tumour cell proliferation^22^; inhibits cadherin 1 expression, resulting in reduced cell‐cell junctions and inducing EMT^23^; binds to the G6PD promoter to activate transcription, which promotes tumour metabolic reprogramming^24^; promotes EMT by reducing hnRNPM expression^25^; and regulates programmed death ligand 1 expression in multiple pathways to facilitate the immune escape of tumour cells.^26^ Previously, YY1 was reported to be aberrantly expressed in PCa and involved in prostate tumorigenesis.^27^, ^28^, ^29^ However, the mechanism underlying YY1 action in inducing malignant proliferation, metastasis, and castration resistance in PCa remains to be further explored.

The results of the current study suggest that ADT upregulates the level of YY1 expression, is involved in PCa cell plasticity, and mediates trans‐differentiation from adenocarcinoma to NE carcinoma via transcriptional activation of FZD8.

## 2 RESULTS

### 2.1 YY1 has an important role in NEPC development

**FIGURE 1:**
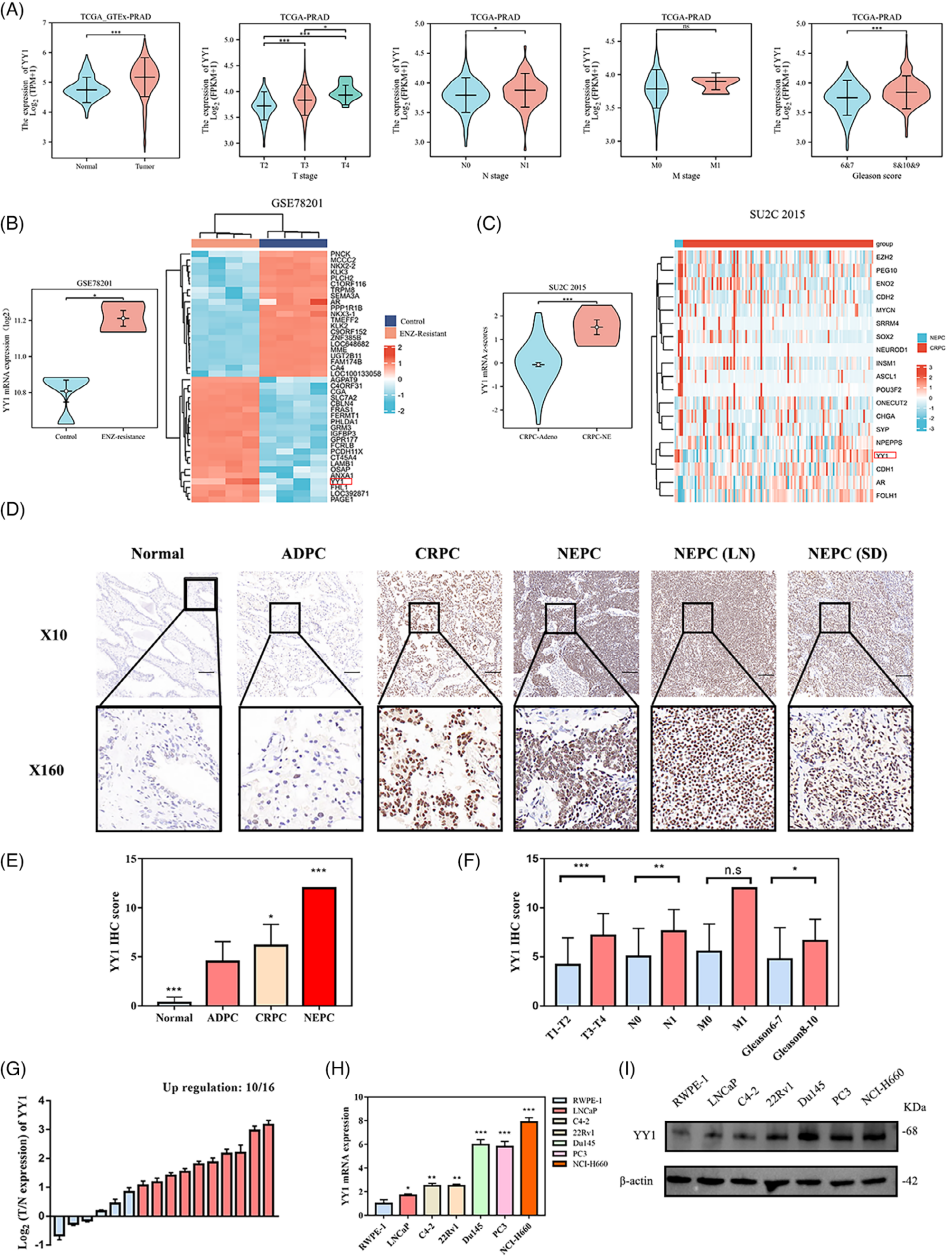
Ying Yang 1 (YY1) had high neuroendocrine prostate cancer (NEPC) expression. (A) Violin plots showing the expression of YY1 in prostate cancer and different clinical variables (T stage, N stage, M stage, and Gleason scores) from the Cancer Genome Atlas (TCGA). (B) Violin plot (left) and heatmap (right) showed the aberrant expression of YY1 in enzalutamide‐resistant prostate cancer (PCa) cells compared to negative control cells from GSE78201. (C) Violin plot (left) and heatmap (right) showed the aberrant expression of YY1 in NEPC compared to castration‐resistant prostate cancer (CRPC) from the SU2C 2015 program. (D) Immunohistochemistry (IHC) staining of YY1 in normal prostate tissue, primary prostate cancer tissue (ADPC), CRPC tissue, NEPC tissue, lymph node (LN) and seminiferous duct (SV) metastasis from NEPC. (E) IHC scores of YY1 in different tissues (normal, ADPC, CRPC, and NEPC). (F) IHC scores of YY1 in different staged prostate cancer tissues. (G) The YY1 mRNA levels in 16 pairs of prostate cancer and matched adjacent normal tissues. Red, log_2_ (tumour:normal expression) value >  1; blue, 1 ≥ log_2_ (tumour:normal expression) value ≥ − 1. (H, I) YY1 expression was examined in RWPE‐1 and PCa cells lines via quantitative reverse transcription polymerase chain reaction (RT qPCR) (H) and western blotting (I). n.s, not significant, *p < .05, **p < .01, ***p < .001.

First, we performed a bioinformatics analysis of the Cancer Genome Atlas (TCGA) dataset to explore the potential role of YY1 in PCa. We found abnormally high levels of YY1 expression in PCa. A significant correlation was demonstrated between YY1 expression and T stage, N stage, and the Gleason score (Figure 1A and Table 1). The aforementioned findings indicated that YY1 has a significant role in the progression and spread of PCa. Nevertheless, the potential involvement of YY1 in enzalutamide resistance and the resulting emergence of NEPC have not been established. GSE78201 was used to scrutinize epigenetic modifications in the acquisition of resistance to enzalutamide in CRPC cells.^30^ The SU2C 2015 program is a potential whole‐exome and transcriptome sequencing database for bone or soft tissue tumour biopsies obtained from 150 metastatic CRPC patients, five of whom had NEPC.^31^ YY1 expression was notably elevated in enzalutamide‐resistant cell lines compared to enzalutamide‐sensitive cell lines, as depicted in Figure 1B. Furthermore, data from the SU2C 2015 program revealed a significant increase in YY1 expression in NEPC samples compared to CRPC samples, as illustrated in Figure 1C. In addition, classical NE factors, such as SOX2, POU3F2 (BRN2), PEG10 and ASCL1, were also shown to be highly expressed in NEPC (Figure S1A). We hypothesize that YY1 contributes significantly to enzalutamide resistance and NEPC. To demonstrate our conjecture, we obtained 55 clinical samples from our center, including primary PCa (*n* = 34), CRPC (*n* = 16), NEPC (*n* = 5), and corresponding adjacent normal prostate tissues, metastatic lymph nodes and vas deferens tissues for immunohistochemical staining (Figure 1D). Based on the immunohistochemistry (IHC) results, YY1 protein is located in the nucleus, is weakly stained in adjacent normal prostate tissues, and has an abnormally high expression in CRPC, especially NEPC (Figure 1E). YY1 protein expression was also shown to be correlated with advanced T stage, N stage, M stage and Gleason score in clinical samples (Figure 1F and Table 2). Interestingly, YY1, SYP and N‐cad staining was more intense in NEPC patients (Figure S1B); the expression was positively correlated in clinical PCa patients (Figure S1C). Furthermore, 16 pairs of clinical PCa tissues and adjacent normal prostate tissues were acquired and subjected to RT‐qPCR analysis. Our findings revealed that the expression of YY1 mRNA was significantly increased in PCa tissues and had a positive correlation with SYP and N‐cad mRNA expression (Figure 1G and Figure S1D). We subsequently observed an upregulation of YY1 mRNA and protein expression in PCa cells, with particularly high levels in Du145 and PC3 cells, which was characterized by an NE‐like phenotype and small cell size (Figure 1H,I).^32^ Overall, our results suggest that YY1 has a crucial role in promoting malignant progression and the development of NEPC in PCa.

**TABLE 1:** Clinical characteristics of prostate adenocarcinoma (PRAD) patients and Ying Yang 1 (YY1) expression in the Cancer Genome Atlas (TCGA) database.

Characteristics	Low expression of YY1	High expression of YY1	p‐Value
n	250	251	
Pathologic T stage, *n* (%)			<.001
T2	113 (22.9%)	76 (15.4%)	
T3	132 (26.7%)	162 (32.8%)	
T4	0 (0%)	11 (2.2%)	
Pathologic N stage, *n* (%)			.153
N0	170 (39.7%)	178 (41.6%)	
N1	32 (7.5%)	48 (11.2%)	
Clinical M stage, *n* (%)			.247
M0	227 (49.3%)	230 (50%)	
M1	3 (0.7%)	0 (0%)	
Gleason score, *n* (%)			.026
6 and 7	159 (31.7%)	135 (26.9%)	
8–10	91 (18.2%)	116 (23.2%)	
Age, *n* (%)			.896
≤60	113 (22.6%)	112 (22.4%)	
>60	137 (27.3%)	139 (27.7%)	
PSA (ng/ml), *n* (%)			.091
<4	209 (47.1%)	208 (46.8%)	
≥4	9 (2%)	18 (4.1%)	

**TABLE 2:** Clinical characteristics of patients and Ying Yang 1 (YY1) expression in our clinical samples.

Characteristics	YY1 expression level		p‐Value
	Low	High	
*n*	24	31	
T stage, *n* (%)			< .001
T1‐2	22 (40%)	6 (10.9%)	
T3‐4	2 (3.6%)	25 (45.5%)	
N stage, *n* (%)			< .001
N0	24 (43.6%)	18 (32.7%)	
N1‐2	0 (0%)	13 (23.6%)	
M stage, *n* (%)			1.000
M0	24 (43.6%)	30 (54.5%)	
M1	0 (0%)	1 (1.8%)	
Pathologic stage, *n* (%)			< .001
I and II	22 (40%)	5 (9.1%)	
III and IV	2 (3.6%)	26 (47.3%)	
Gleason score, *n* (%)			< .001
6 and 7	19 (34.5%)	10 (18.2%)	
≥8	5 (9.1%)	21 (38.2%)	
Age, mean ± standard deviation	68.333 ± 7.423	72.097 ± 6.6451	.053

### 2.2 YY1 promoted PCa progression in vitro

**FIGURE 2:**
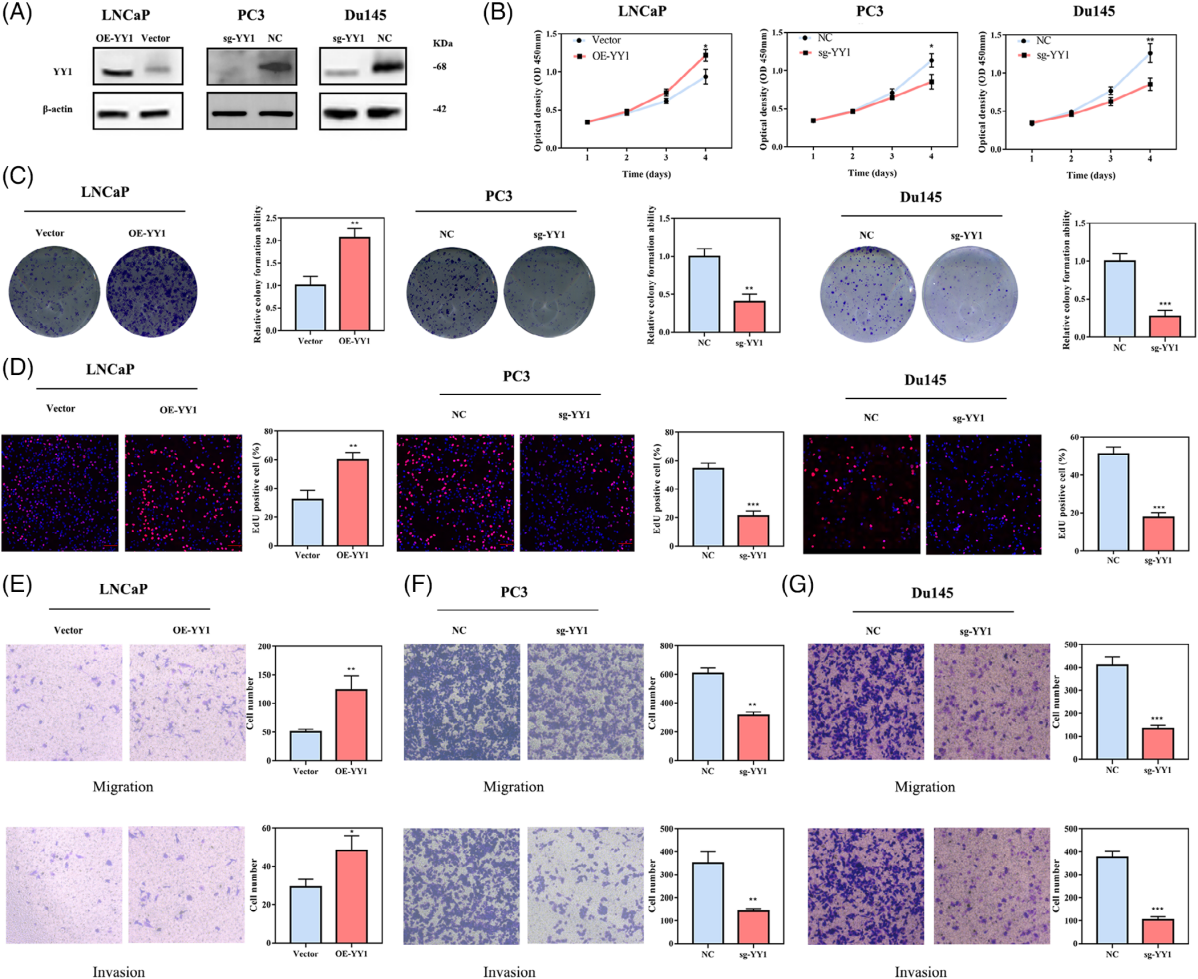
Ying Yang 1 (YY1) promoted prostate cancer (PCa) progression in vitro. (A) Efficiencies of YY1 inhibition and overexpression were examined by western blotting. (B–D) The effects of YY1 inhibition and overexpression on cell proliferation, detected by Cell Counting Kit‐8 (CCK‐8) (B), clone formation (C) and EdU assays (D). (E) The effects of YY1 overexpression on migration (above) and invasion (below) were observed in LNCaP by Transwell assays. (F, G) The effects of YY1 knockout on cellular migration (above) and invasion (below) observed in PC3 (F) and Du145 cells (G) by Transwell assays. *p < .05, **p < .01, ***p < .001.

Given that NEPC is a more aggressive type of PCa, our aim was to determine if YY1 is involved in tumour proliferation, invasion, and migration. YY1 expression analysis led us to overexpress and knockout YY1 by transfecting OE‐YY1 lentivirus into LNCaP cells, and CRISPR/Cas9 YY1 knockout in PC3 and Du145 cells, respectively (Figure 2A). Overexpression of YY1 promoted LNCaP cell growth. In contrast, knockout of YY1 inhibited PC3 and Du145 cell growth, as determined by the Cell Counting Kit‐8 (CCK‐8) assay (Figure 2B). Using the clone formation assay we then demonstrated the promotion of YY1 overexpression on the formation of clones in LNCaP cells, which was inhibited upon YY1 knockout in PC3 and Du145 (as depicted in Figure 2C). (Figure 2D). Our subsequent objective was to determine the role of YY1 in regulating tumour metastasis through cellular migration, invasion and wound healing assays. The findings indicated that overexpression of YY1 facilitated the migration and invasion of LNCaP cells (Figure 2E). Conversely, YY1 knockout resulted in a significant reduction in the migration and invasion of PC3 and Du145 cells, as shown in Figure 2F,G. Additionally, YY1 knockout led to a significant decrease in the wound healing capacity of PC3 and Du145 cells (Figure S2A,B). Furthermore, the role of YY1 in regulating cancer cell stemness was investigated using the sphere formation assay, which revealed that YY1 overexpression increased the number and volume of LNCaP cells, while YY1 knockout had a significant inhibitory effect (Figure S2C,D).

### 2.3 YY1 promoted PCa cell growth and metastasis in vivo

**FIGURE 3:**
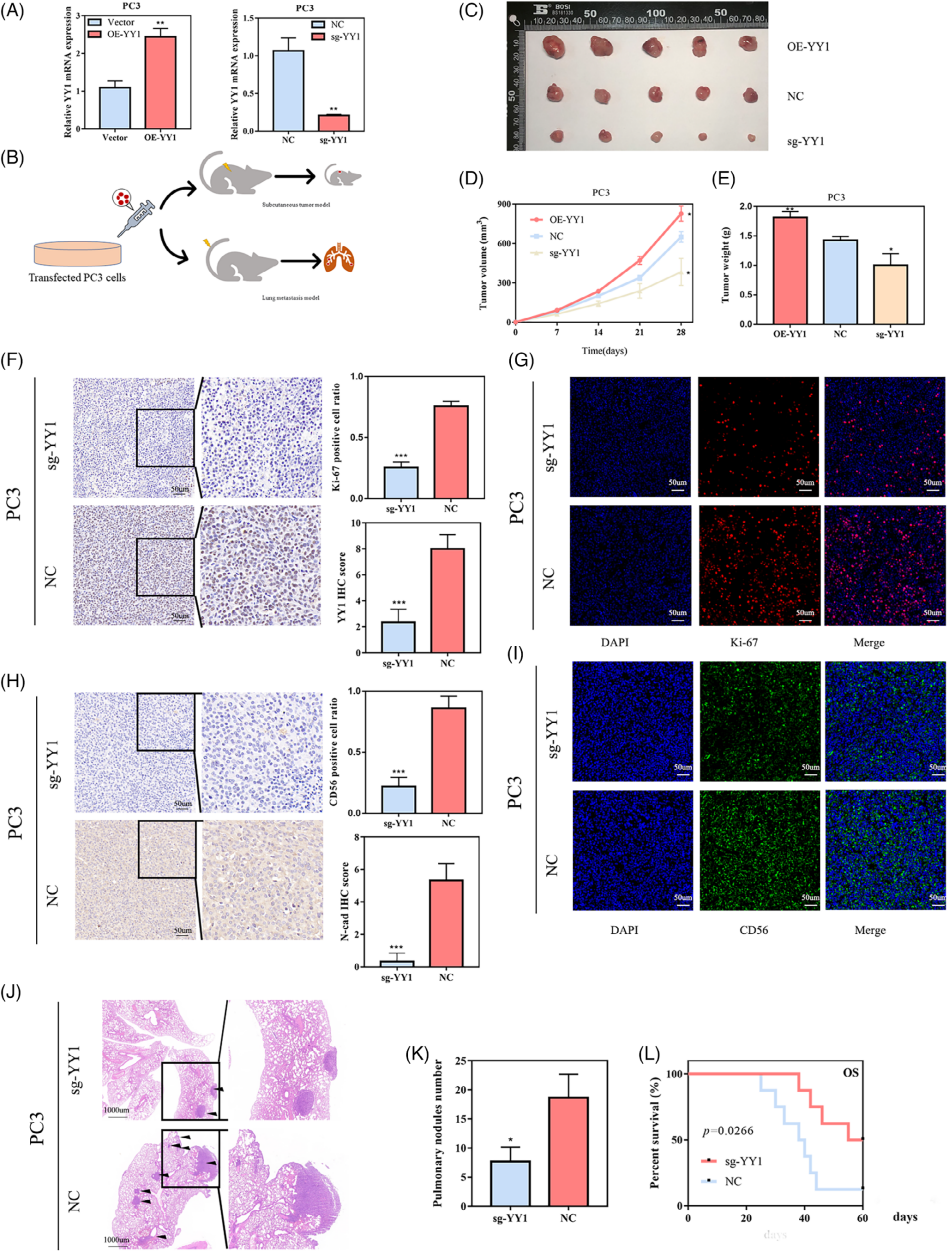
Ying Yang 1 (YY1) promoted prostate cancer (PCa) cell growth and metastasis in vivo. (A) Quantitative reverse transcription polymerase chain reaction (RT qPCR) was used to verify the transfection efficiency of YY1. (B) Graphical illustration of two in vivo mouse model studies. (C–E) Tumour size (C, D) and weight (E) of the subcutaneous tumour growth model injected with transfected PCa cells (5 mice/group). (F) Immunohistochemistry (IHC) analysis (left) and IHC score (right) for YY1 of subcutaneous tumour sections. (G) immunofluorescence analysis (right) and quantitative bar graph (left) for Ki‐67 of subcutaneous tumour sections. (H) IHC analysis (left) and IHC score (right) for N‐cad of subcutaneous tumour sections. (I) Immunofluorescence analysis (right) and quantitative bar graph (left) for CD56 of subcutaneous tumour sections. (J, K) The effects of YY1 knock out on lung metastasis (J) and the number of metastases (K) were examined by hematoxylin‐eosin staining (H&E) staining. (L) Overall survival analysis of mice in lung metastasis *p < .05, **p < .01. T, tumour; N, normal.

Figure 3A illustrates the efficiency of YY1 overexpression and knockout in PC3 cells. Graphical illustrations of two in vivo mouse model studies are depicted in Figure 3B. A subcutaneous injection model was used to examine YY1 involvement in tumour growth in vivo. Transfected PCa cells were injected subcutaneously into nude mice, and the results showed that YY1 overexpression stimulated the growth of subcutaneous tumours. Conversely, the YY1 suppression significantly impeded tumour growth (Figure 3C–E). We collected the xenograft tumours and performed IHC and immunofluorescence (IF) assays to further identify the role of YY1 in vivo. The IHC results of xenograft tumours showed that YY1 staining was reduced by YY1 knockout (Figure 3F). The IF results showed a significantly lower proportion of Ki‐67‐positive cells in the YY1 knockout xenograft tumours than the negative control group, suggesting that YY1 contributed to PCa cell proliferation in vivo (Figure 3G). In addition, we observed N‐cad expression via IHC and showed that YY1 knockout reduced N‐cad expression in xenograft tumours (Figure 3H). A cell surface glycoprotein (CD56) regulates embryogenesis, development and neural cell connectivity. CD56‐positive cancers, such as small‐cell lung carcinoma, mesothelioma, carcinoid tumours, islet cell tumours and tumours with NE differentiation, are more aggressive than CD56‐negative cancers.^33^, ^34^, ^35^ The number of CD56‐positive cells was also less in the sg‐YY1 group than the control group (Figure 3I). Moreover, our lung metastasis models demonstrated that YY1 knockout inhibited the aggressiveness of PCa cells in vivo. Hematoxylin‐eosin staining (H&E) staining revealed smaller metastatic lesions (Figure 3J) and fewer pulmonary metastatic nodules in the YY1 knockout group (Figure 3K). Survival analysis showed that the YY1 knockout group had longer overall survival (Figure 3L). Both in vitro and in vivo data suggested that YY1 contributed to NEPC proliferation, migration, and invasion.

### 2.4 Upregulation of YY1 induced EMT and NE trans‐differentiation in PCa cells

**FIGURE 4:**
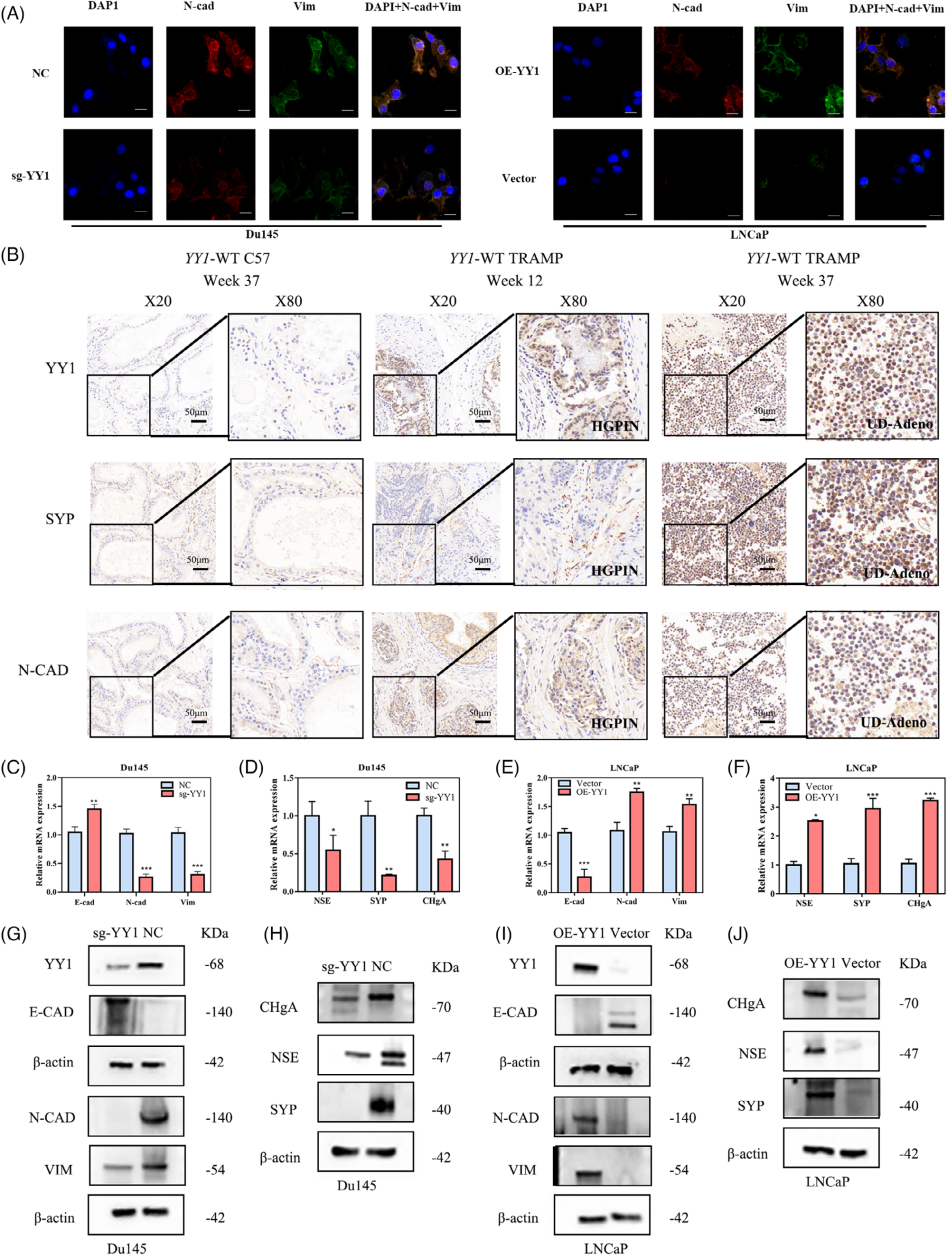
Ying Yang 1 (YY1) upregulation induced epithelial‐mesenchymal transition (EMT) and neuroendocrine differentiation in prostate cancer (PCa) cells. (A) The expression and location of vimentin (Vim) and N‐cadherin (N‐cad) markers in Du145‐NC/Du145‐sg‐YY1 and LNCaP‐Vector/LNCaP‐OE‐YY1 cells were analyzed by immunofluorescence staining. (B) The immunohistochemistry (IHC) staining of murine prostatic lesions at weeks 12 and 37. (C–F) Quantitative reverse transcription polymerase chain reaction (RT qPCR) analyses show EMT (N‐cadherin, vimentin and E‐cadherin) and neuroendocrine (NE) marker expression (chromogranin A [CHgA], neuron‐specific enolase [NSE] and synaptophysin [SYP]) in Du145‐NC/Du145‐sg‐YY1 (C, D) and LNCaP‐vector/LNCaP‐OE‐YY1 (E, F) cells. (G–J) Western blotting shows EMT and NE marker expression in Du145‐NC/Du145‐sg‐YY1 (G, H) and LNCaP‐Vector/LNCaP‐OE‐YY1 (I, J) cells. *p < .05, **p < .01, ***p < .001.

The ability of PCa to migrate and invade, and the degree of resistance to treatment, varies across the cell lineages from adenocarcinoma to NE trans‐differentiation. Therefore, we determined whether YY1 mediated malignancy progression in PCa by promoting cell plasticity. The results of the assays demonstrated that YY1 upregulation in LNCaP cells (AR‐positive) led to the induction of mesenchymal markers (N‐cad and Vim). Conversely, YY1 suppression in Du145 cells (AR‐negative) resulted in a significant reduction in the expression of mesenchymal markers (Figure 4A). These findings suggested that YY1 overexpression endowed adenocarcinoma cells (LNCaP) with a mesenchymal cell phenotype, while YY1 knockout induced mesenchymal‐epithelial transition (MET) in CRPC cells. The TRAMP mouse model has been extensively used in PCa research due to the ability to accurately mimic the pathogenesis of PCa in humans.^36^, ^37^ The TRAMP model exhibits a progression from prostatic intraepithelial neoplasia (PIN; 10–12 weeks of age) to aggressive prostate adenocarcinoma (18–20 weeks of age) via the genetic expression of viral SV40 cancer protein in prostate epithelial cells.^38^ Eventually, approximately 20% of TRAMP mice will develop NEPC.^39^ According to previous studies,^40^ undifferentiated adenocarcinoma (UD‐adeno) lesions are classified as NEPC lesions based on NEPC‐like characteristics, such as pleomorphism, high nuclear‐to‐cytoplasmic ratios, and NEPC‐like characteristics in cells. In the current study, IHC staining of prostate tissues from 37‐week‐old control C57 and 12‐ and 37‐week‐old YY1‐WT TRAMP mice showed that YY1 protein expression increased with age in TRAMP mice, and was highest in UD‐adeno samples, suggesting that YY1 may be essential for NE trans‐differentiation (Figure 4B). Subsequent quantitative reverse transcription polymerase chain reaction (RT qPCR) and western blotting assays further confirmed that YY1 overexpression induced LNCaP cells to lose the epithelial phenotype and acquire the NE and mesenchymal phenotypes, while YY1 knockout promoted Du145 cells to undergo MET and lose the NE phenotype (Figure 4C–J). Furthermore, the reduction in NE marker expression (Figure S3A), inhibition of NCI‐H660 cell proliferation, and suppression of subcutaneous tumour growth in vitro and in vivo (Figure S3B–D) were observed upon YY1 knockout in NCI‐H660 (NEPC cells). Additionally, construction of a lung metastasis model using Tramp C1 cells derived from TRAMP mice transfected with sg YY1/NC revealed a significant decrease in the number of metastatic lung nodules (Figure S3E,F). Taken together, these findings suggest that YY1 may serve as a crucial regulator of cellular plasticity in PCa, thereby facilitating the progression of NEPC.

### 2.5 ADT inhibited YY1 ubiquitination and promoted plasticity in PCa cells

**FIGURE 5:**
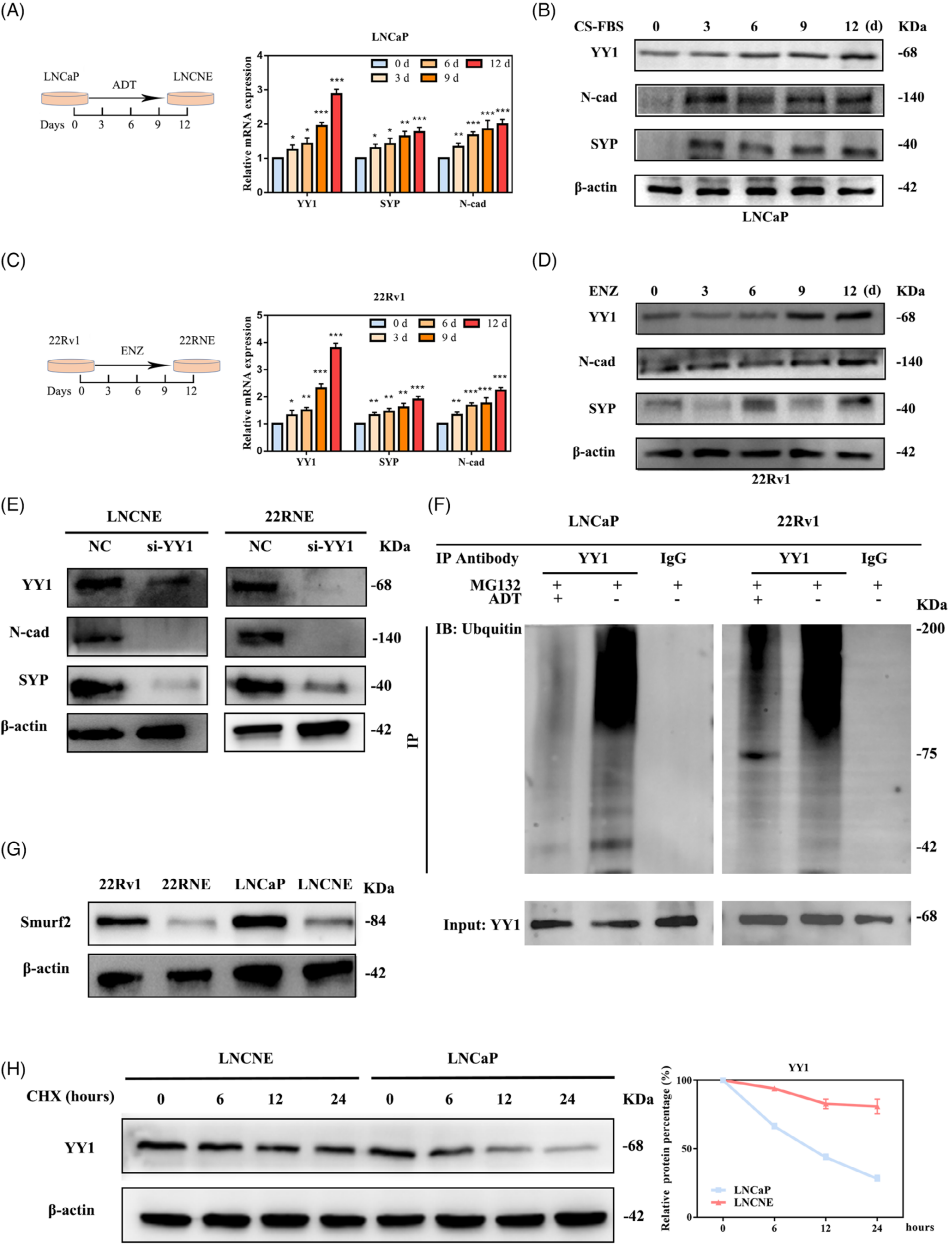
Androgen deprivation therapy (ADT) inhibited Ying Yang 1 (YY1) ubiquitination and promoted plasticity in prostate cancer (PCa) cells. (A) Culture model of LNCaP cells in charcoal‐stripped fetal bovine serum (CS‐FBS) (left); quantitative reverse transcription polymerase chain reaction (RT qPCR) analyses show YY1, N‐cadherin, and SYP at different time points (right). (B) Western blotting was used to detect YY1, N‐cadherin, and SYP expression in LNCaP cells under CS‐FBS for the indicated times. (C) Culture model of 22Rv1 cells in enzalutamide (left); RT qPCR analyses showed YY1, N‐cadherin, and SYP expression at different time points (right). (D) Western blotting was used to detect YY1, N‐cadherin, and SYP expression in 22Rv1 cells under enzalutamide for the indicated time. (E) Western blotting was used to observe YY1, N‐cadherin, and SYP expression in LNCNE and 22RNE cells after YY1 knockdown. (F) Impact of ADT‐induced neuroendocrine differentiation of LNCaP and 22Rv1 cells on YY1 ubiquitination. Cell lysates of neuroendocrine prostate cancer (NEPC) and PCa cells were immunoprecipitated with anti‐YY1 and analyzed by western blotting. (G) Cells were subjected to western blotting to detect the level of Smurf2 protein. (H) LNCaP and LNCNE cells were treated with 10 μg/mL of cycloheximide (CHX) for the indicated times and harvested for western blotting. n.s, not significant, *p < .05, **p < .01.

The terminal stage of PCa (NEPC) occurs following ADT.^12^, ^41^ Upon culturing in phenol red‐free, RPMI‐1640 medium with 10% charcoal‐stripped fetal bovine serum (CS‐FBS), the LNCaP cells gradually acquired an NE phenotype (Figure S4A,B), corroborating previous studies.^42^, ^43^ In addition, ADT enhanced NE differentiation and EMT induced by OE‐YY1 in LNCaP cells (Figure S4C,D). We therefore investigated whether ADT induced PCa cell plasticity via YY1. LNCaP and 22Rv1 cells were cultured in CS‐FBS and enzalutamide for 12 days, respectively, to establish in vitro ADT models at different time points. We found that YY1, NE marker (SYP) and mesenchymal marker (N‐cad) expression increased gradually with time after ADT treatment (Figure 5A–D). Furthermore, we demonstrated reversed SYP and N‐cad expression upon YY1 depletion in LNCaP and 22Rv1‐derived NEPC, indicating that ADT induced PCa cell plasticity by upregulating YY1 expression (Figure 5E and Figure S4E). To further investigate the effect of AR inactivation on YY1 protein expression, LNCaP cells were stimulated with dihydrotestosterone (DHT; Solarbio) after ADT to determine changes in YY1 expression. We showed that YY1 expression was decreased and NE dedifferentiation occurred after DHT treatment (Figure S5A). EZH2 could methylate histone H3 lysine 27 (H3K27) and altering the expression of lineage‐specific genes is a well‐recognized feature of NEPC.^44^, ^45^ Consequently, we performed a series of assays to determine the potential involvement of EZH2 in YY1‐mediated plasticity. Our findings indicated that EZH2 suppression resulted in a decrease in SYP and N‐cad expression, while YY1 expression remained unchanged in NEPC cells. Interestingly, SYP and N‐cad expression in OE‐YY1 LNCaP cells was not significantly affected by knockdown of EZH2 (Figure S5B,C). This finding suggests that EZH2 may not be involved in regulating YY1‐induced cellular plasticity. In addition, we found lower YY1 ubiquitination in LNCaP and 22Rv1‐derived NEPC compared to control PCa cells (Figure 5F). Smurf2 is the specific E3 YY1 ubiquitination ligase, which has been reported in previous studies.^46^ It is noteworthy that Smurf2 expression was lower in aggressive PCa and exhibited a negative correlation with YY1 expression (Figure S5D–F). This observation suggested that Smurf2 inhibition may be responsible for the low level of YY1 ubiquitination in NEPC. To further explore the regulatory role of Smurf2 in YY1, knockdown of Smurf2 in PCa cells was shown to increase YY1 protein expression and decrease YY1 ubiquitination, while having no effect on the level of YY1 mRNA (Figure S5G–I). In agreement with the above results, we showed that the level of Smurf2 protein was reduced after ADT compared to control cells (Figure 5G), and the level of YY1 protein gradually decreased in LNCaP cells but remained unchanged in LNCNE cells (Figure 5H). Together, these findings suggested that ADT reduced YY1 ubiquitination and thus modulated cellular plasticity in PCa.

### 2.6 FZD8 is the downstream target of YY1

**FIGURE 6:**
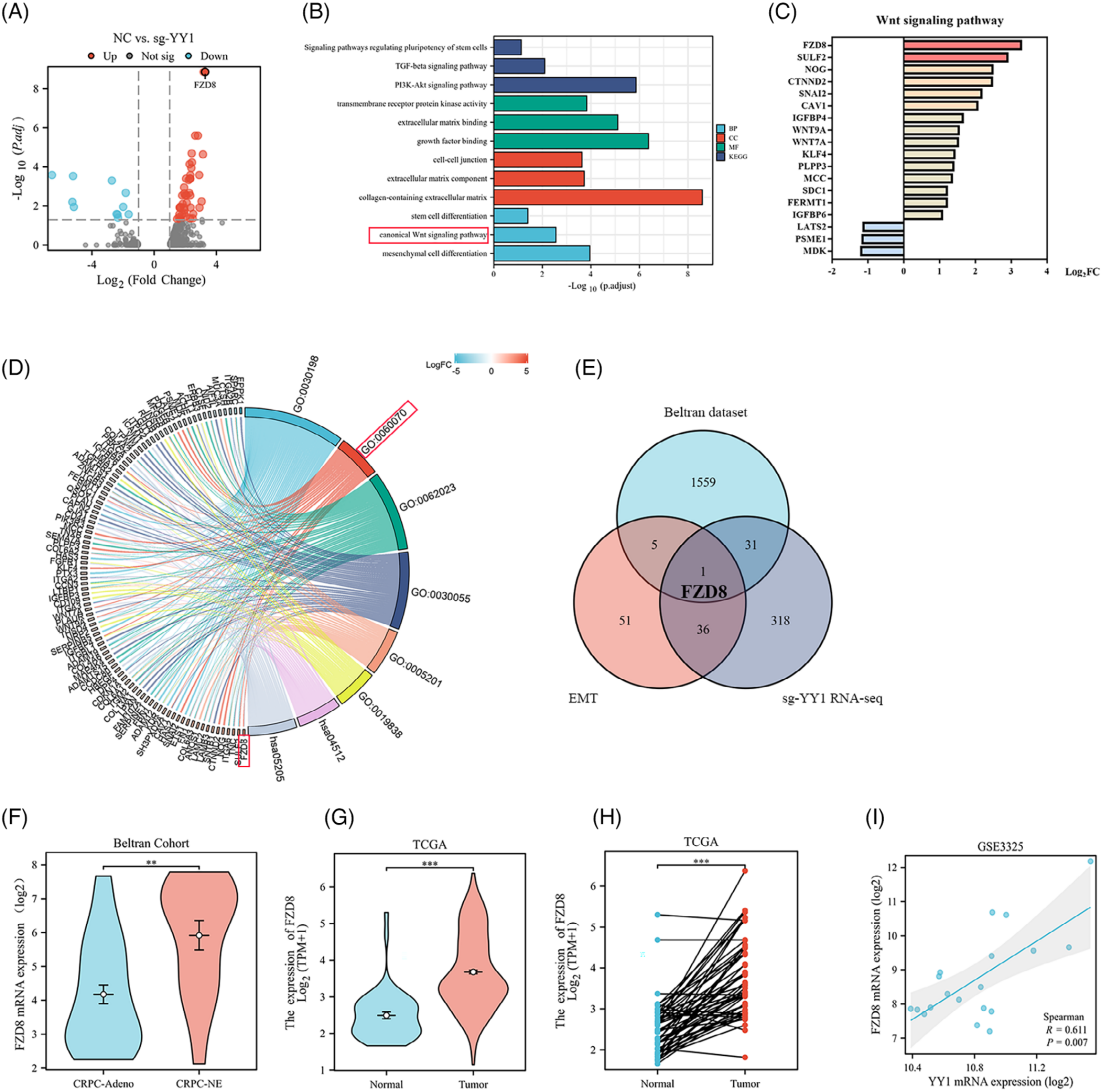
FZD8 is the downstream target of Ying Yang 1 (YY1). (A) Volcano plots showing the differentially expressed genes identified by RNA sequencing (RNA‐seq) in PC3‐sg‐YY1 cells compared to control cells. Cut‐off values were identified with an adjusted p‐value < .05 and log_2_ (|fold change|)  >  1. Red, YY1 positively related genes; blue, YY1 negatively associated genes. (B) Gene Ontology (GO) and Kyoto Encyclopedia of Genes and Genomes (KEGG) functional pathway analyses of YY1 target genes. (C) Differentially expressed downstream target YY1 genes enriched in the Wnt signaling pathway. (D) GOChord plot of the functional interaction network of target YY1 genes. (E) Venn diagram showing the potential downstream target of YY1 involved in cell plasticity. Cut‐off values were identified with a p‐value < .05 and log_2_ (|fold change|)  >  1 for sgYY1 RNAseq; a p‐value < .05 and fold change  >  1.5 for Beltran datasets. (F) Level of FZD8 expression in Beltran datasets. (G, H) Level of FZD8 expression in non‐paired (G) and paired (H) prostate cancer samples in the Cancer Genome Atlas (TCGA) dataset. I Co‐expression analysis of YY1 and FZD8 in GSE3325. The correlation coefficient was calculated by Spearman analysis. **p < .01, ***p < .001.

To determine the mechanism by which YY1 is involved in NEPC progression, we performed RNA sequencing (RNA‐seq) analysis on YY1 knockout in PC3 cells to identify the downstream target genes of YY1. The volcano plot revealed the identification of mRNAs that were differentially expressed with adjusted *p*‐values < .05 and log_2_ (|fold change|) > 1 (Figure 6A and Table S1). To investigate the biological functions and pathways affected by these differentially expressed genes (DEGs), Gene Ontology (GO) and Kyoto Encyclopedia of Genes and Genomes (KEGG) analyses were performed. The results revealed significant enrichment of the following terms: “mesenchymal cell differentiation;” “extracellular matrix components;” “stem cell development;” “neural crest cell differentiation;” and “Wnt signaling pathway” (Figure 6B and Figure S6A–D). We also performed gene set enrichment analysis (GSEA) with DEGs, which showed the “epithelial‐mesenchymal transition” signaling pathway to be significantly enriched (Figure S6E,F). The above results collectively implied that upon YY1 knockout, the DEGs mainly exhibited enrichment of EMT‐related signaling pathways (Figure 6C). The GOChord plot demonstrated the functional interaction network of differential genes (Figure 6D). To identify the key downstream YY1 genes regulating the plasticity of PCa cells, we intersected genes from YY1 knockout DEGs, YY1‐related EMT genes and Beltran dataset NE‐related DEGs (fold change > 1.5; *p* < .05). Notably, we showed that FZD8 was involved in EMT and NE differentiation (Figure 6E and Table S2). Moreover, the results from the Beltran dataset and GSE3325 showed that FZD8 expression was upregulated in primary PCa, especially in metastatic PCa and NEPC (Figure 6F–H and Figure S6G). Furthermore, a significant positive correlation was observed between FZD8 and YY1 expression in GSE3325 (Figure 6I). These findings were consistent with our subsequent investigation of FZD8 expression in PCa cell lines, which revealed a significant upregulation of FZD8 in DU145 and PC3 cells (Figure S6H). To further elucidate the role of FZD8 in cell migration, we performed experiments involving the transfection of a plasmid overexpressing FZD8 in LNCaP and the knockdown of FZD8 in Du145 and PC3 cells. Our results indicated that FZD8 overexpression promoted LNCaP migration, while FZD8 knockdown inhibited migration and wound healing in PC3 and Du145 cells (Figure S6I,J). In addition, FZD8 knockdown reduced mesenchymal and NE marker expression (Figure S6K). In conclusion, FZD8 is involved in regulating PCa migration as well as cell plasticity.

**FIGURE 7:**
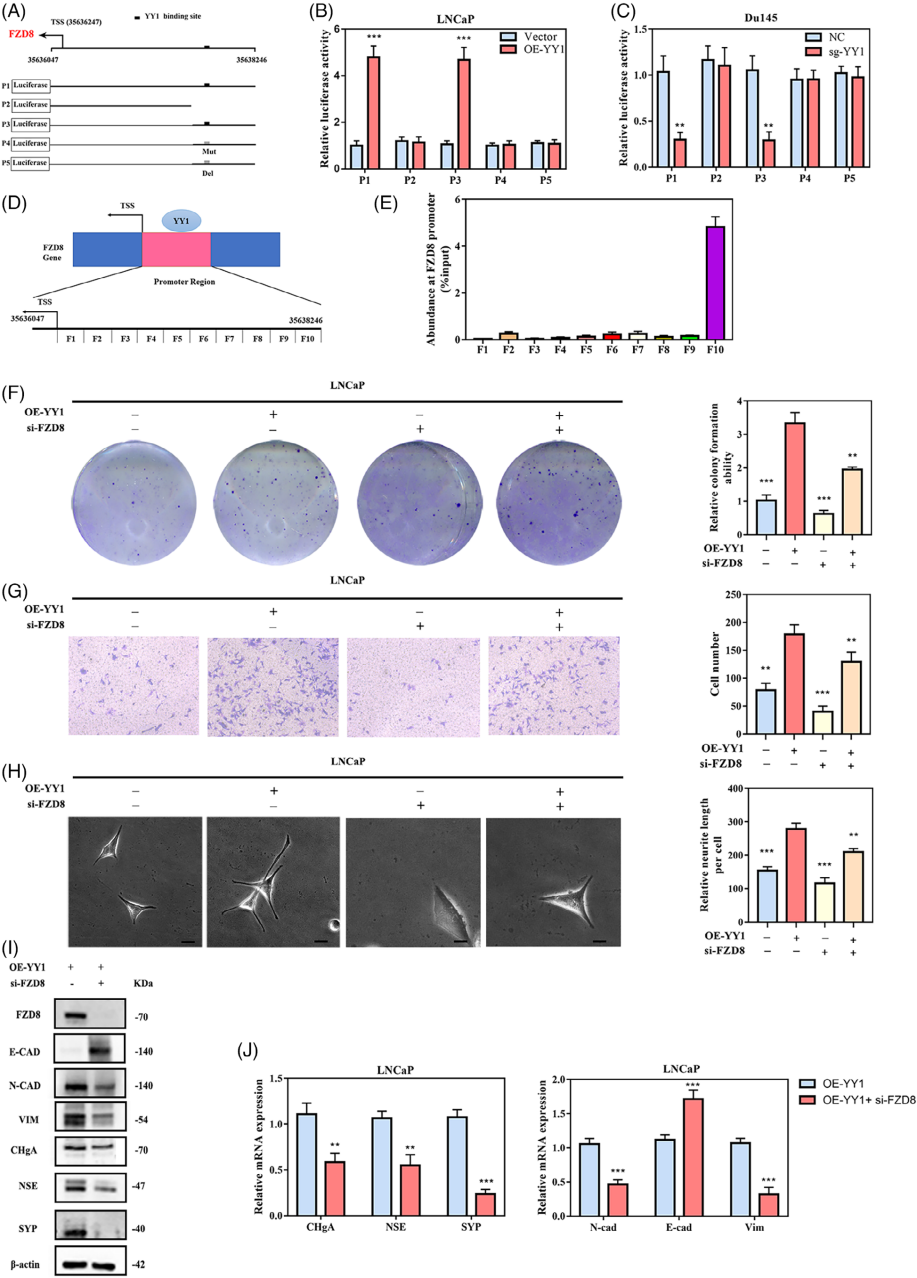
FZD8 is a functional mediator of Ying Yang 1 (YY1) in regulating cell plasticity. (A) Five designed serial FZD8 promoter fragments for the luciferase assay. TSS, transcriptional start site. (B, C) Luciferase assays showed the different transcriptional activity of wt‐YY1 on the serial FZD8 promoter fragments, as indicated in LNCaP (B) and Du145 (C) cells. (D, E) In a Chromatin IP (ChIP) assay, wt‐YY1 binds to YY1‐specific binding sites at the FZD8 promoter. (D), the FZD8 promoter fragments (F1–10) in the precipitated DNA were amplified by PCR (E) with primers specific to these fragments. (F–H) Rescue experiments showing the effect of YY1 overexpression in LNCaP cells could be counteracted by FZD8 inhibition in colony formation assays (F), Transwell assays (G), and neurogenesis assays (H). (I, J) Western blotting (I) and quantitative reverse transcription polymerase chain reaction (RT qPCR) (J) analyses showed the level of epithelial‐mesenchymal transition (EMT) and neuroendocrine (NE) marker expression of YY1 overexpression in LNCaP cells could be counteracted by FZD8 inhibition. n.s, not significant, *p < .05, **p < .01, ***p < .001.

YY1 is a transcription factor causing transcriptional activation/inhibition by binding directly to the promoter of a downstream gene,^47^, ^48^ leading us to investigate YY1‐mediated transcriptional regulation of FZD8 expression. In fact, YY1 overexpression in LNCaP upregulates FZD8 at the mRNA and protein levels. In contrast, the YY1 knockout in Du145 cells resulted in downregulation of FZD8 mRNA and protein levels compared to the control (Figure S7A,B). UCSC and PROMO database analyses of the FZD8 promoter region revealed YY1‐specific binding sites, suggesting probable FZD8 transcriptional regulation by YY1 (Figure 7A). Moreover, the YY1 overexpression in LNCaP increased FZD8 promoter‐driven luciferase activity, suggesting transcriptional regulation (Figure 7B). In contrast, the YY1 knockout in Du145 cells produced the opposite result (Figure 7C). However, FZD8 promoters containing mutated or deleted binding sites for YY1 failed to affect luciferase activity independent of YY1 overexpression or knockout (Figure 7B,C). In addition, we further performed Chromatin IP (ChIP) experiments, which showed that YY1 was bound to a specific region of the FZD8 promoter (Figure 7D,E). Moreover, ADT‐induced FZD8 promoter luciferase activity in LNCaP was reversed by YY1 knockdown. These results indicated that ADT induced the transcriptional activity of FZD8 via YY1 in PCa cells (Figure S7C).

### 2.7 FZD8 is a functional mediator of YY1 in the regulation of PCa cell plasticity

Next, we knocked down FZD8 in the OE‐YY1 LNCaP or negative control cells to determine if YY1 involved regulation of FZD8 (Figure S7D). The findings indicated that the proliferation, migration, and NE differentiation of LNCaP cells were significantly reversed by FZD8 knockdown in response to YY1 overexpression, as depicted in Figure 7F–H. Furthermore, upregulation of mesenchymal and NE markers in OE‐YY1 LNCaP cells was reversed by FZD8 knockdown, as shown in Figure 7I,J. These results suggested that YY1 modulated the plasticity of PCa cells by upregulating FZD8.

### 2.8 YY1 regulated PCa cell plasticity via the non‐canonical Wnt pathway

**FIGURE 8:**
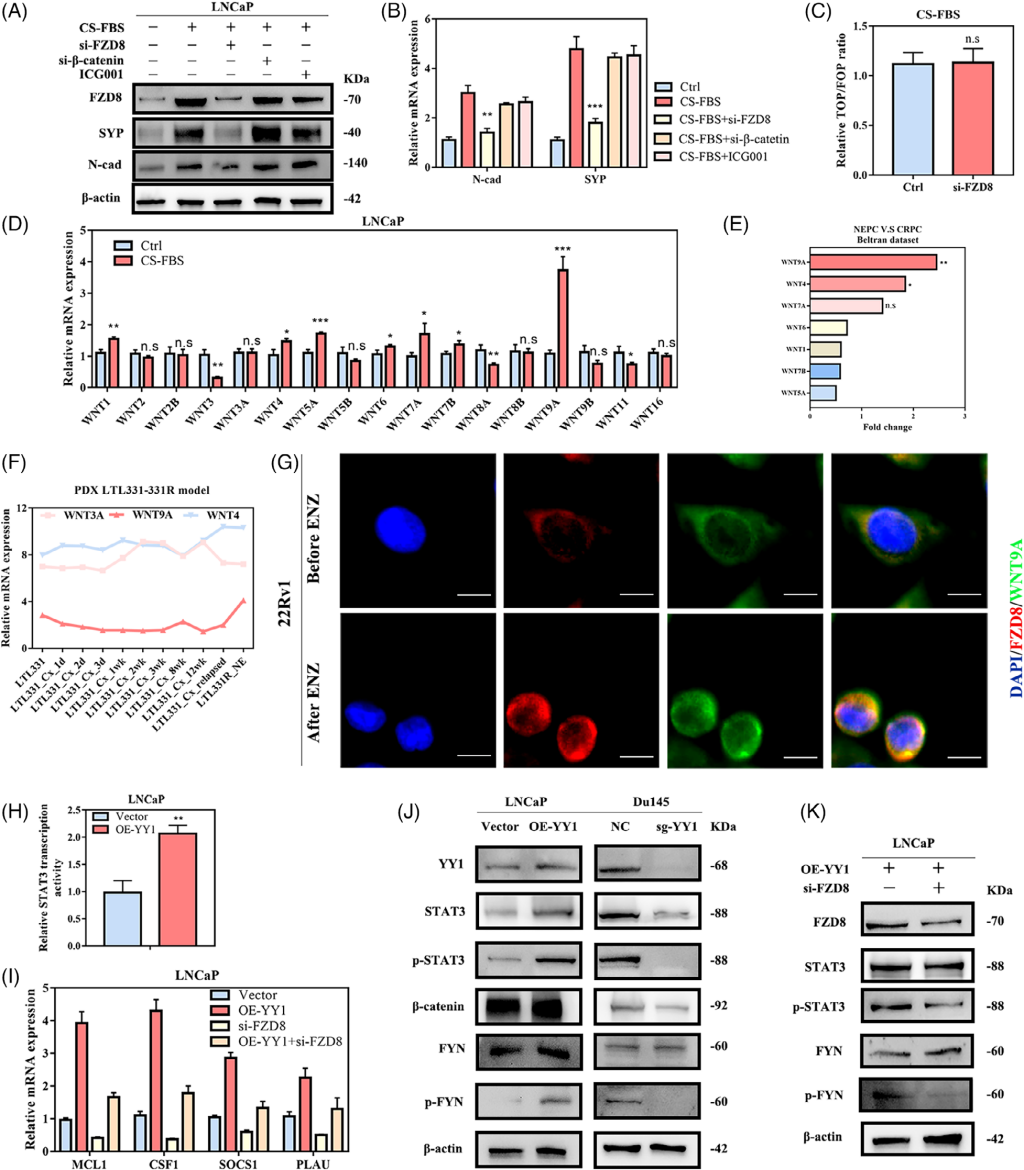
Ying Yang 1 (YY1) regulated prostate cancer cell plasticity through a non‐canonical Wnt pathway. (A, B) Western blotting (A) and quantitative reverse transcription polymerase chain reaction (RT qPCR) (B) analyses were used to observe the role of depleted FZD8 and β‐catenin on charcoal‐stripped fetal bovine serum (CS‐FBS)‐induced cell plasticity in LNCaP cells. (C) Luciferase assay showing the role of FZD8 knockdown on the β‐catenin/TCF‐dependent transcriptional activity. (D) RT qPCR analyses show the level of Wnt ligand mRNA after CS‐FBS treatment. (E) Beltran datasets show the fold‐change of indicated Wnt ligands in neuroendocrine prostate cancer (NEPC) versus castration‐resistant prostate cancer (CRPC). (F) LTL331‐331R patient‐derived xenograft (PDX) model demonstrates changes in mRNA levels of the indicated Wnt ligands with castration time series. (G) Immunofluorescence assays showing the sub‐localization of FZD8 and Wnt9A in prostate cancer (PCa) cells and the binding effect after androgen deprivation therapy (ADT) treatment. (H) Luciferase assay showing the role of YY1 overexpression on the transcriptional activity of STAT3. (I) RT qPCR analyses were performed to observe the effect of depleted FZD8 after YY1 overexpression on STAT3 downstream genes. (J) Western blot was used to detect the activity of FYN and STAT3 signalling pathways in Du145‐NC/Du145‐sg‐YY1 and LNCaP‐Vector/LNCaP‐OE‐YY1 cells. (K) Western blotting showing the activity of FYN and STAT3 signalling pathways in LNCaP‐OE‐YY1 cells after FZD8 knockdown. n.s, not significant, *p < .05, **p < .01, ***p < .001.

FZD8, a member of the frizzled family, is a receptor for the Wnt pathway,^49^ and reportedly involved in EMT regulation in PCa cells by activating β‐catenin‐dependent (canonical) and ‐independent (non‐canonical) Wnt pathways.^50^, ^51^ Therefore, we aimed to determine if ADT‐induced EMT and NE differentiation depended on the canonical Wnt/β‐catenin pathway. Our results showed that FZD8, a mesenchymal (N‐cad) and NE marker (SYP), were upregulated in LNCaP cells after ADT (Figure 8A). We used siRNA‐mediated silencing of FZD8 and β‐catenin, and β‐catenin inhibitors (ICG001) to determine the effects on ADT‐induced cell plasticity. Depletion of FZD8, but not β‐catenin, effectively counteracted ADT‐induced cell proliferation, migration, and NE differentiation of PCa cells (Figure S8A–C). It is noteworthy that FZD8 knockdown significantly reversed N‐cad and SYP upregulation induced by ADT, while depletion of β‐catenin had no effect on this phenomenon (Figure 8A,B). Furthermore, silencing of FZD8 did not impede β‐catenin/TCF‐dependent transcriptional activity in LNCNE cells (Figure 8C). Therefore, we considered that induction of PCa lineage plasticity under androgen deficiency may be mediated by non‐canonical Wnt signaling.

We performed RT qPCR analysis to identify the Wnt ligands that are upregulated in NEPC and found that Wnt9A, Wnt7A, and Wnt4 were upregulated in LNCaP cells after ADT (Figure 8D). In addition, we also investigated the differential expression of Wnt ligands in the Beltran dataset, which revealed that Wnt9A expression was 2.5‐fold higher, followed by a 1.8‐fold increase of Wnt4 in NEPC compared to CRPC (Figure 8E). Additionally, patient‐derived xenograft tissue was shown to induce Wnt ligand expression during castration.^52^ LTL331 xenografts exhibiting a classic prostate adenocarcinoma phenotype were transformed into castration‐resistant NEPC LTL331R tumours, during which Wnt9A and Wnt4 was markedly upregulated (Figure 8F). Further investigations revealed that FZD8 expression in PCa was only positively correlated with Wnt9A expression (Figure S8D,E). IF analysis showed that FZD8 and Wnt9A were primarily distributed in cell membranes. Moreover, binding of FZD8 to Wnt9A was significantly enhanced under ADT, which induced Wnt9A upregulation and binding to FZD8 (Figure 8G). Interfering with signaling processes that activate the EMT and cancer stem cells programs, which maintain the phenotype that confers cellular plasticity, prevented the emergence of NEPC. Interleukin (IL)‐6‐STAT3 signalling is one of the most well‐characterized of these signaling pathways.^9^ In the current study, GSEA analysis also indicated that downstream YY1 genes were enriched in the IL6‐STAT3 signaling pathway (Figure S8F). This result was also confirmed by CS‐FBS treatment after which ADT activated the FYN/STAT3 pathway in LNCaP cells (Figure S8G). To further clarify the role of Wnt9A in promoting cell plasticity, we transfected overexpressed Wnt9A plasmids (p‐Wnt9A) into LNCaP cells and performed western blot analysis. Wnt9A overexpression activated STAT3 and upregulated SYP and N‐cad expression (Figure S8H). In addition, knockdown of Wnt9A (si‐Wnt9A) or a Wnt secretion inhibitor (C59) reduced SYP and N‐cad expression in OE‐YY1 LNCaP cells (Figure S8I). In contrast, overexpression of Wnt9A facilitated YY1‐induced NE plasticity (Figure S8J).

In the current study, we further explored the role of the STAT3 signaling pathway in YY1‐mediated cellular plasticity. YY1 regulated the transcriptional activity of STAT3 and mediated STAT3 downstream target gene expression via FZD8 in LNCaP cells (Figure 8H,I). Transfection of si‐STAT3 or use of a STAT3 inhibitor (stattic) in LNCaP cells overexpressing YY1 downregulated N‐cad and SYP expression (Figure S9A). Moreover, STAT3 transcriptional activation by exogenous IL‐6 treatment was approximately 2–3‐fold higher in LNCaP or Du145 cells (Figure S9B). Our experiments revealed that ruxolitinib, a JAK1/2 inhibitor, blocked exogenous IL‐6‐induced STAT3 activation. However, JAK inhibition had a minimal effect on YY1‐induced STAT3 transcription in LNCAP or Du145 cells (Figure S9B). Taken together, these data suggested that YY1‐mediated cellular plasticity via activation of the STAT3 signaling pathway, which may be independent of IL6‐induced Janus kinase activity. Therefore, a search for bypass activation pathways independent of IL6‐induced Janus kinase is warranted.

FYN, a downstream target of the receptor tyrosine kinase pathway, activates the STAT3 signaling pathway.^53^, ^54^ Previous studies also reported that FZD2 binds to the SH2 domain of FYN, and thus activates STAT3 to regulate the non‐canonical Wnt pathway.^55^ Moreover, Dishevelled (DVL) potentially mediates β‐catenin‐independent signaling.^56^ To determine whether there is an interaction among FZD8, DVL and FYN in the cellular environment, we performed co‐immunoprecipitation (CO‐IP) experiments with Du145 cells. We detected the interaction of FZD8 with DVL and FYN (Figure S9C). In addition, our investigations revealed that YY1 activated STAT3 and FYN signaling in PCa cells without upregulation of β‐catenin (Figure 8J), while depletion of FZD8 in OE‐YY1 LNCaP cells led to inactivation of STAT3 and FYN signaling (Figure 8K). Moreover, STAT3 activation, and SYP and N‐cad expression were observed in LNCaP cells transfected with a plasmid overexpressing FZD8 (p‐FZD8; Figure S9D). The above findings indicated that FZD8 is the key mediator for YY1 in the regulation of FYN/STAT3 signaling. To determine the role of FYN/STAT3 in cellular plasticity, we transfected si‐STAT3 and si‐FYN into PCa cells or treated the cells with the corresponding inhibitors to observe the changes mesenchymal and NE marker expression. We showed that FYN or STAT3 knockdown significantly reduced the levels of mesenchyme and NE marker expression in p‐FZD8 LNCaP cells (Figure S9E). In addition, we treated p‐FZD8 LNCaP and Du145 (NE‐like cells with high FZD8 expression) with stattic or the FYN inhibitor (dasatinib) and showed that dasatinib inhibited the activities of FYN and STAT3 and reversed NE plasticity, while stattic inactivated STAT3 and reduced SYP and N‐cad expression (Figure S9F–I). FZD8 knockdown or administration of C59 significantly decreased the phosphorylation levels of STAT3 and FYN in LNCNE cells (Figure S9J), while FYN knockdown reduced the level of STAT3 phosphorylation, suggesting that FYN is a functional mediator of STAT3 pathway activation by FZD8. In addition, FZD8 knockdown or administered with C59 in Du145 cells significantly suppressed STAT3 transcriptional activity (Figure S9K). In summary, we considered that YY1 promotes cell lineage conversion by modulating the FYN/STAT3 signaling pathway through FZD8.

## 3 DISCUSSION

Despite effective treatments based on ADT to inhibit the AR signaling pathway being successful, treatment resistance is almost universal.^57^ A subset of therapy‐resistant patients exhibit the NE phenotype, a lethal disease characterized by low prostate‐specific antigen (PSA) levels and plastic trans‐differentiation of adenocarcinoma to NE carcinoma.^9^ Indeed, YY1 is highly expressed in advanced PCa, particularly NEPC. ADT inhibits YY1 ubiquitination to prevent its degradation and promotes PCa cell plasticity towards NE trans‐differentiation via the STAT3 signaling pathway.

Atchison et al.^57^ questioned the oncogenic status of YY1 because few studies have been reported on the carcinogenic effects of YY1 in in vivo and in vitro experiments. The promoter region of YY1 is characterized by a high guanine‐to‐cytosine (G:C) content, particularly in the 5′ untranslated region (5′ UTR) of the mRNA. This feature facilitates the formation of a G‐quadruplex (G4) structure within the YY1 promoter and 5′ UTR, akin to proto‐oncogenes, such as c‐Myc and Bcl‐2.^58^, ^59^ It has been reported that YY1 is aberrantly expressed in most tumours, including PCa.^60^ The current investigation showcases that the anomalous manifestation of YY1 stimulates the proliferation, migration, and invasion of PCa cells in vitro, and triggers the growth of tumours as well as metastasis to the lungs in vivo. Recent studies on breast,^61^ gastric,^62^ and colon cancers^63^ have also confirmed the carcinogenic effects of YY1 in vitro and in vivo.

PCa cells exhibit variable differentiation potential after ADT. The transmembrane protein, N‐cadherin, is essential in the EMT of cancer cells because N‐cadherin facilitates cell‐cell adhesion and migration.^64^ Moreover, inhibition of AR signaling leads to CRPC by upregulation of EMT drivers (ZEB1, ZEB2, Snail and Twist) and mesenchymal markers (N‐cadherin).^65^ In the current study ADT‐induced, AR‐positive PCa cells (LNCaP) acquired a mesenchymal phenotype (N‐cadherin). The same result was demonstrated in Du145 cells (NE‐like PCa cells). Introducing a new generation of AR pathway inhibitors has significantly benefitted the survival of CRPC patients for an extra 10 months.^66^ However, ARs can be reactivated via AR splice variants, AR activating mutations and AR amplifications, leading to treatment resistance.^9^ Abiraterone/enzalutamide provides no additional survival benefits to mCRPC patients.^67^ Another mechanism fueling treatment resistance is manifested as AR‐independent PCa, which is characteristic of small‐cell carcinoma and NEPC.^68^ In the present study ADT enabled LNCaP and 22Rv1 cells to acquire an NE phenotype and to express NE markers (SYP). Therefore, we hypothesized that inhibition of the AR signaling pathway causes PCa cells to undergo lineage plasticity trans‐differentiation, and eventually acquire therapeutic resistance.

Dysregulated transcription factors, such as FOXA1, FOXA2, SOX2 and ONECUT2, have a critical role in NEPC progression.^32^, ^69^, ^70^, ^71^ We showed that the zinc finger transcription factor, YY1, which is upregulated upon ADT treatment, activates FZD8 transcription and is involved in cellular plasticity. ADT also induced post‐transcriptional modifications that inhibited activation of Smurf2 and contributed to stabilization of YY1 protein. The critical role of YY1 in NEPC was further confirmed by IHC analysis of patient‐derived NEPC tissue and TRAMP mice.

FZD8, a constituent of the frizzled (FZD) protein family, functions as a receptor for WNT ligands. Prior studies indicated that FZD8 is overexpressed in diverse tumours, resulting in bone metastasis and resistance to treatment by activating the canonical Wnt/β‐catenin pathway.^51^, ^72^, ^73^ In the current study, we identified FZD8 as a critical downstream gene activated by YY1 via promoter binding. An enhanced binding effect of FZD8 to Wnt9A after ADT was observed in PCa cell membranes based on IF assay results, thus contributing to PCa cell plasticity by activating a non‐canonical Wnt pathway (FZD8/FYN/STAT3).

## 4 CONCLUSION

In summary, YY1 is another crucial transcription factor inducing NE differentiation in PCa that promotes tumour cell growth and metastasis in vivo and in vitro. ADT was shown to enhance YY1 protein stability, which mediates cell plasticity via a non‐canonical Wnt (FZD8/FYN/STAT3) signaling pathway.

## 5 METHODS AND MATERIALS

### 5.1 Data availability

The microarray datasets and prostate adenocarcinoma transcriptome were acquired from the Gene Expression Omnibus (GEO [https://www.ncbi.nlm.nih.gov/geo/]), cBioPortal (http://www.cbioportal.org/) and TCGA databases (https://portal.gdc.cancer.gov/), respectively, to assess the level of YY1 expression in various PCa samples. The GSE78201^30^ dataset, which includes untreated and enzalutamide‐treated PCa cell lines, is based on the GPL10558 platform. RNA‐seq data for the NEPC clinical cohort were obtained from Beltran et al.^41^ and SU2C 2015 program.^31^

### 5.2 Cell cultures

The human PCa cell lines, LNCaP, Du145 (NE‐like cell), PC3 (NE‐like cell), C4‐2, 22Rv1, NCI‐H660 (small cell NECP derived from lymph node metastasis) and normal prostate epithelial cells (RWPE‐1), were procured from the American Type Culture Collection cell bank. The cells were maintained at 37°C in a 5% CO_2_ humidified atmosphere and cultured in media containing 10% FBS (Gibco) and 1% penicillin/streptomycin. RPMI‐1640 (Gibco) was used to culture LNCaP, Du145, PC3, 22Rv1 and C4‐2, while DMEM/F12 was used for NCI‐H660. The NE phenotype was induced by culturing cells in phenol red‐free RPMI‐1640 medium (Gibco) supplemented with 10% CS‐FBS^42^ for 1 week. Neurite length in LNCaP cells was quantified by analyzing five random fields of view using the Fiji plugin Simple Neurite Track following the guidelines provided by the manufacturer.^74^

### 5.3 Cell transfection

A CRISPR/Cas9 gene knockout system for YY1 (sg‐YY1) was constructed and validated by GeneChem (Shanghai, China). Both the interfering RNAs (siRNAs) and overexpression lentivirus (OE‐YY1) were designed by GenePharma (Shanghai, China). Lentivirus for YY1 overexpression and lentiviral vectors were transfected into cells according to the protocol, and the lentiviral vectors were used as negative controls. The FZD8 and WNT9A plasmids were synthesized and validated by Corues Biotechnology. In addition, siRNAs and plasmids were transfected via the jetPRIME transfection reagent. Transfection efficiency was verified by RT qPCR and western blotting. The target sequences of siRNAs and lentivirus are shown in Table S3.

### 5.4 RT qPCR, western blotting and IP

The RNAeasy Animal RNA Isolation Kit (Beyotime) was utilized to extract total RNA from cultured cells and tissues, which was subsequently reverse‐transcribed to cDNA with the SweScript cDNA Synthesis SuperMix (Servicebio) in accordance with the manufacturer's instructions. The expression of mRNAs was normalized using GAPDH. Total protein lysates were extracted using RIPA (Beyotime) and quantified using a BCA Protein Assay Kit (KeyGEN). An equivalent amount of protein was separated by 6%–12% SDS‐PAGE and transferred onto PVDF membranes. After blocking, membranes were incubated overnight with primary antibody at 4°C, followed by 2 h with secondary antibody at room temperature. In the end, Bio‐Rad ChemiDoc XRS (Hercules) was used to visualize bands.^75^ An IP kit (Beyotime) was utilized for IP assays. The antibodies were preincubated with magnetic beads, followed by incubation with the cell lysate supernatant overnight at 4°C. The beads were subsequently washed, and SDS‐PAGE sample loading buffer (1X) was added and heated at 95°C for 5 min. The supernatant obtained after separating the beads was utilized for western blotting. The primer sequences and antibody information are listed in Tables S3 and S4.

### 5.5 Chromatin IP

According to the manufacturer's instructions, a Simple ChIP Enzymatic Chromatin IP Kit with magnetic beads (Cell Signaling TECHNOLOGY) was used for ChIP. A list of the ChIP primers is presented in Table S3.

### 5.6 Colony formation, CCK‐8 and EdU assays

The experimental protocol involved seeding cells into 96‐well plates in triplicate for the CCK‐8 (Dojindo Laboratories), followed by measurement of cell viability in accordance with the manufacturer's instructions. Colony formation was assessed by seeding cells into 6‐well plates (500 cells/well) in triplicate and culturing for 10 days, after which the colonies were fixed, stained, and counted. For the EdU assay kit (RiboBio), cells were seeded into 24‐well plates (3 × 10^4^ cells/well) in triplicate, cultured for 24 h, then incubated with 50 μM EdU for 2 h. Subsequent staining and visualization were performed.^76^ The EdU assay involves the addition of a penetrant to cell climbing slides that have been prepared and subsequently incubated in a rocker device for 10 min. Subsequently, the EdU staining reaction solution was added and incubated at room temperature for 30 min in the dark. After three washes with phosphate‐buffered saline (PBS), the slides were incubated for 10 min at room temperature with DAPI solution (Servicebio). The liquid was then discarded, and the slide was covered with a fade‐resistant mounting solution. Finally, the images were collected and examined using fluorescence microscopy.

### 5.7 Cell migration, invasion and sphere formation assay

Transwell inserts (Corning) were utilized to perform migration and invasion assays in accordance with the manufacturer's guidelines. The migrated cells were enumerated and captured via a light microscope. For the sphere formation assay, 500 cells were seeded onto 6‐well ultra‐low cluster plates (Corning) and cultured in DMEM/F12 medium supplemented with 20 ng/ml of bFGF (PeproTech), 2% B27 (Life Technologies), and 20 ng/ml of EGF (PeproTech). Following a 10‐day incubation period, the spheres were quantified and imaged.

### 5.8 Promoter activity and TOP/FOP luciferase reporter assays

The Luciferase Assay System (Promega) was used to perform luciferase reporter assays in accordance with the manufacturer's instructions. Specifically, transfection of FZD8 promoters with mutated or deleted YY1‐specific binding sites or the TOPFlash/FOPFlash plasmids into cells was carried out using a transfection reagent (Vazyme Biotech). Subsequently, luciferase activity was measured 48 h post‐transfection using a Dual‐Luciferase Reporter Assay Kit (Promega), with normalization based on the Renilla luciferase signal. Information on the predicted YY1 binding site to the FZD8 promoter is provided in Table S5.

### 5.9 IF assay and IHC

We performed IF assays to detect gene expression and cellular co‐localization. Briefly, we incubated the cells with primary antibodies at 4°C overnight, followed by incubation with the corresponding secondary antibodies and DAPI. Images were captured using a confocal microscope. The IHC method was described in detail in a previous study.^75^ In summary, the human PCa and animal model tissues were fixed, embedded and sectioned prior to staining for IHC. The sections were subsequently dried at 60°C for 2 h and incubated with primary and secondary antibodies overnight at 4°C and for 1 h at room temperature, respectively. After DAB visualization, hematoxylin was utilized for counterstaining, followed by dehydration in graded alcohol, condensation in xylene and covering with Permount for microscopic observation. Table S4 contains all relevant information pertaining to the antibodies utilized in both IF and IHC.

### 5.10 Animal models

The present study used a subcutaneous model involving male BALB/c nude mice 4–6 weeks old for investigating tumour growth. Specifically, 5 × 10^6^ transfected cells were suspended in 100 μl of PBS and subcutaneously injected into the right mid‐posterior axilla of the nude mice. Tumour volumes were measured on a weekly basis, and after 5 weeks the mice were euthanized and the tumour weights were recorded. Subsequently, subcutaneous tumours were collected for further analysis via IF and IHC staining. To establish a lung metastases model, 1 × 10^6^ transfected cells were injected into the tail vein of each mouse. The lung tissues from each mouse were collected and stained with H&E after 60 days of tumour development. Pulmonary metastases were counted and survival curves were plotted.

The experimental use of transgenic TRAMP+ mice, which were derived from C57BL/6 male mice obtained from the Jackson Laboratory. Prostatic tissue samples were collected from two age groups (12 weeks [*n* = 5 per group] and 37 weeks [*n* = 5 per group]) and classified by pathologists based on histologic features as normal tissues, high‐grade PIN (HGPIN) and UD‐adeno.

### 5.11 Statistical analysis

The experiments were conducted in triplicate. A single representative experiment has been included in the manuscript. The data were analyzed using mean ± standard deviation and statistical differences were evaluated using Student's t‐test or two‐way analysis of variance. Immunohistochemical scores were utilized to assess clinicopathologic features. Survival comparisons between groups were made using Kaplan‐Meier survival curves and log‐rank tests. Statistical analysis and plotting were performed using R software (version 4.2.0) or GraphPad Prism (version 7.0). Statistical significance was considered as follows: *p < .05; **p < .01; and ***p < .001.

## CONFLICT OF INTEREST STATEMENT

The authors declare no conflict of interest.

## FUNDING INFORMATION

This study was funded by The National Natural Science Foundation of China (Nos. 81872089, 81370849, 81672551, 81300472, 81070592, 81202268 and 81202034).

## Supporting information

Supporting Information

Table S1. RNA‐seq analysis on YY1 knockout PC3 cells.

Table S2. Venn results.

Table S3. The information of primers, siRNAs, overexpression/knockdown lentivirus.

Table S4. The information of antibodies.

Table S5. Information on the predicted binding site and motifs of YY1 to the FZD8 promoter.

## Data Availability

Datasets used and/or analyzed during this study can be accessed on reasonable request from the corresponding author.
