# Supplementary material for: Yin Yang 1 promotes the neuroendocrine differentiation of prostate cancer cells via the non‐canonical WNT pathway (FYN/STAT3)
Source: Clin Transl Med. 2023 Sep 28;13(10):e1422. doi: 10.1002/ctm2.1422 (PMC10539684; doi:10.1002/ctm2.1422)
Supplement: Supplementary file 1 — Supporting Information [file CTM2-13-e1422-s001.docx]

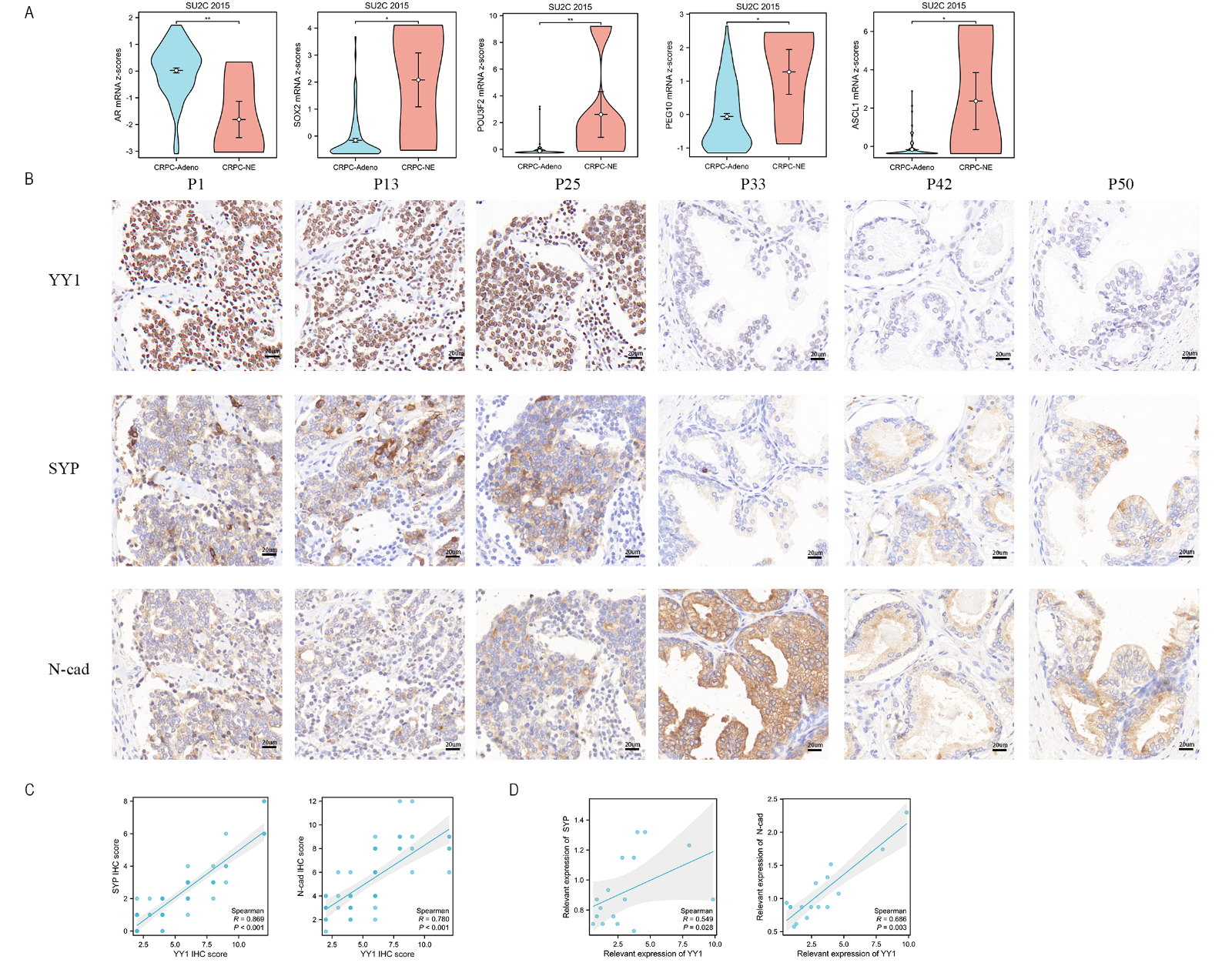


**Figure S1. YY1 expression in prostate cancer. A.** Expression profile of some NE-related genes in the SU2C 2015 program. **B.** YY1, SYP, and N-cad IHC analysis of prostate cancer tissue sections from clinical PCa patients. **C.** Correlation analysis of IHC scores between YY1, and SYP and N-cad. **D**. mRNA co-expression analysis of YY1, and SYP and N-cad in prostate cancer tissues. *p < 0.05, **p < 0.01, ***p < 0.001


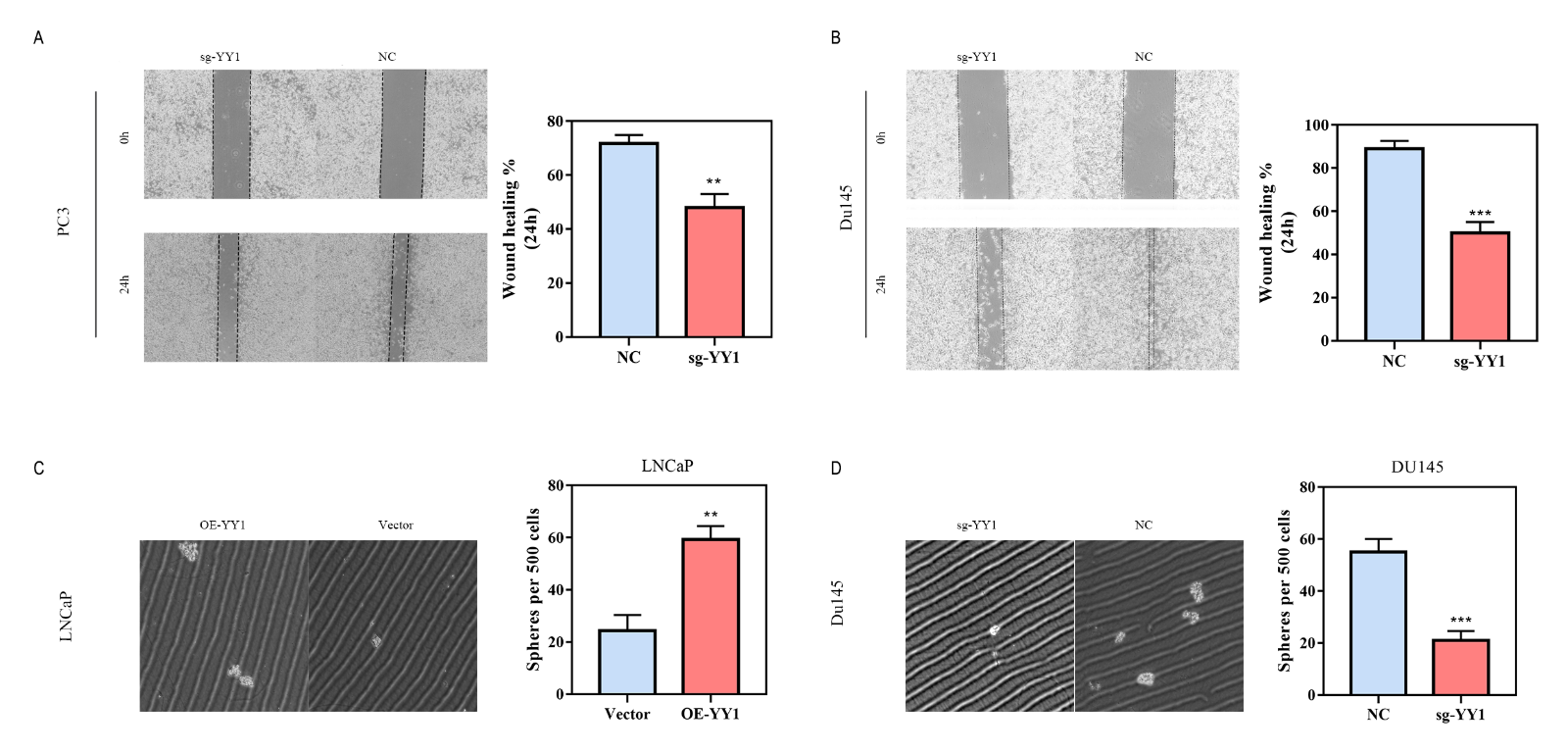


**Figure S2. YY1 promoted migration and sphere formation in prostate cancer cells. A-B.** Wound healing assays showed knockout of YY1 inhibited the migration of PC3 cells (**A**) and Du145 cells (**B**). **C-D.** Overexpression of YY1 in LNCaP cells promoted sphere formation (**C)**, knockout of YY1 in Du145 cells inhibited sphere formation. *p < 0.05, **p < 0.01, ***p < 0.001


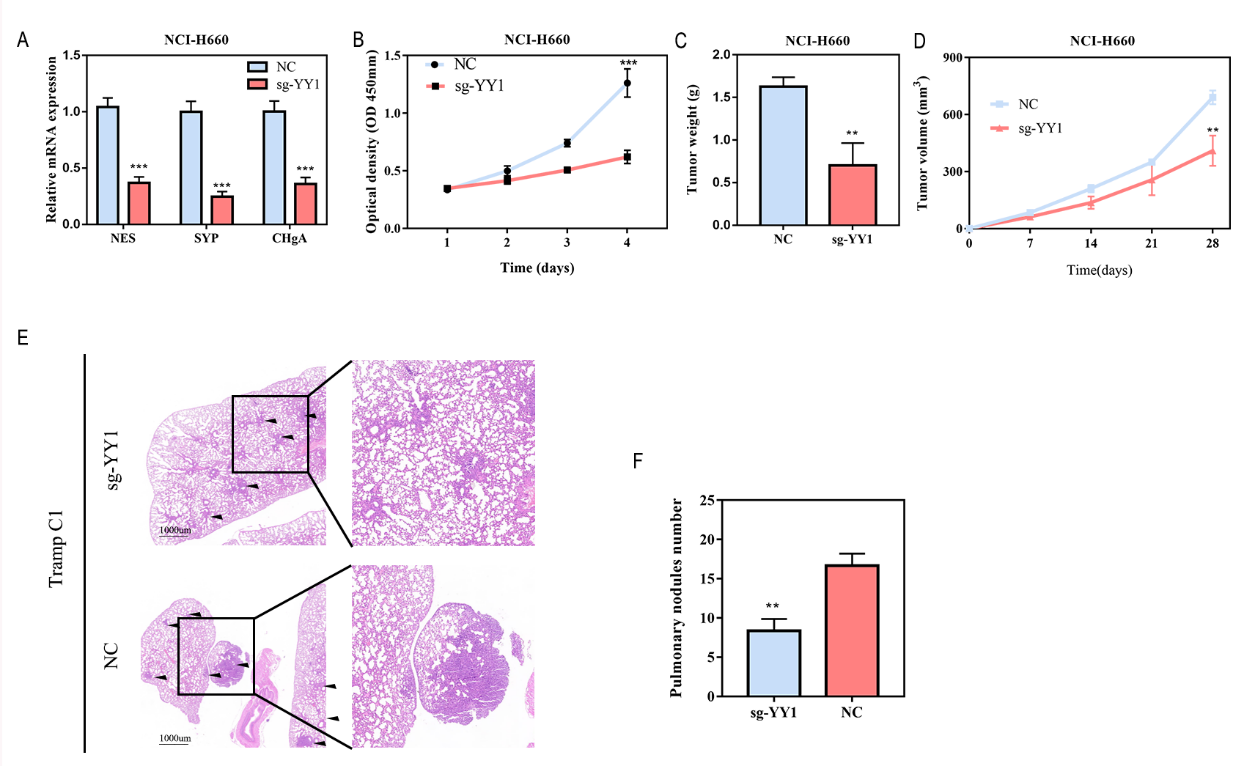


**Figure S3. YY1 knockout assays in NEPC and the Tramp C1 cell line.** **A.** RT qPCR analyses were used to detect the expression and NE markers in NCI-H660-NC/NCI-H660-sg-YY1 cells. **B.** CCK-8 was used to observe the effects of YY1 inhibition on cell proliferation in NEPC. **C, D.** Tumor weight (**C**) and size (**D**) of the subcutaneous tumor growth model injected with transfected NCI-H660 cells (5 mice/group). **E-F.** Tramp C1 cells transfected with sg-YY1/NC were injected into the tail vein of mice to observe lung metastasis (**E**), Knockout YY1 inhibited lung metastasis (**F**). **p < 0.01, ***p < 0.001


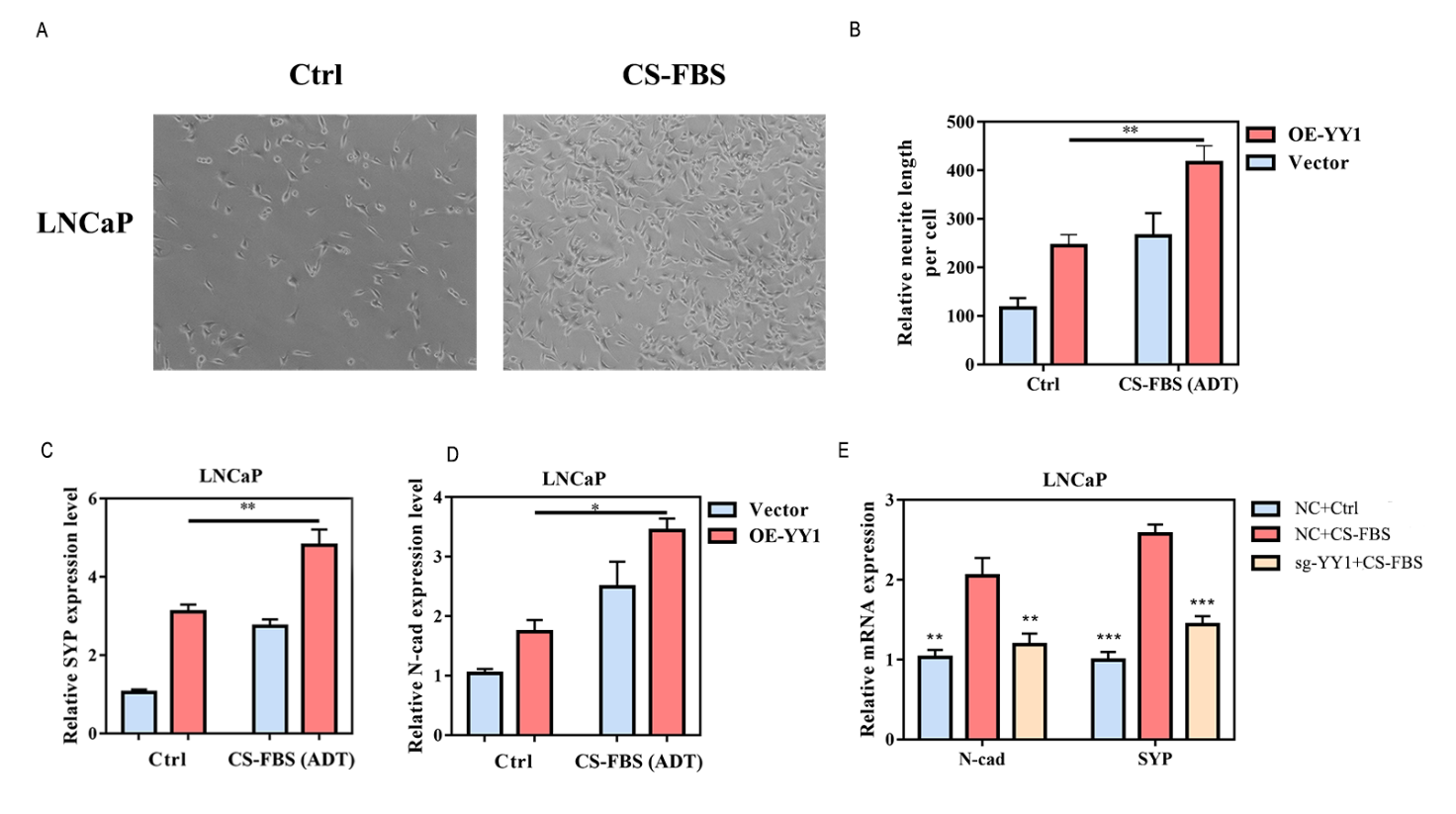


**Figure S4. CS-FBS induced plasticity in prostate cancer cells via YY1.** **A.** CS-FBS induced neuroendocrine differentiation in LNCaP cells. **B.** Neurogenesis analysis in LNCaP cells with and without overexpression of YY1 under CS-FBS treatment. **C, D.** RT qPCR analyses were used to examine SYP (**C**) and N-cad expression (**D**) in LNCaP cells with and without overexpression of YY1 under CS-FBS treatment. **E.** RT qPCR analyses were used to examine SYP and N-cad expression in LNCaP cells with and without knockout of YY1 under CS-FBS treatment. *p < 0.05, **p < 0.01, ***p < 0.001


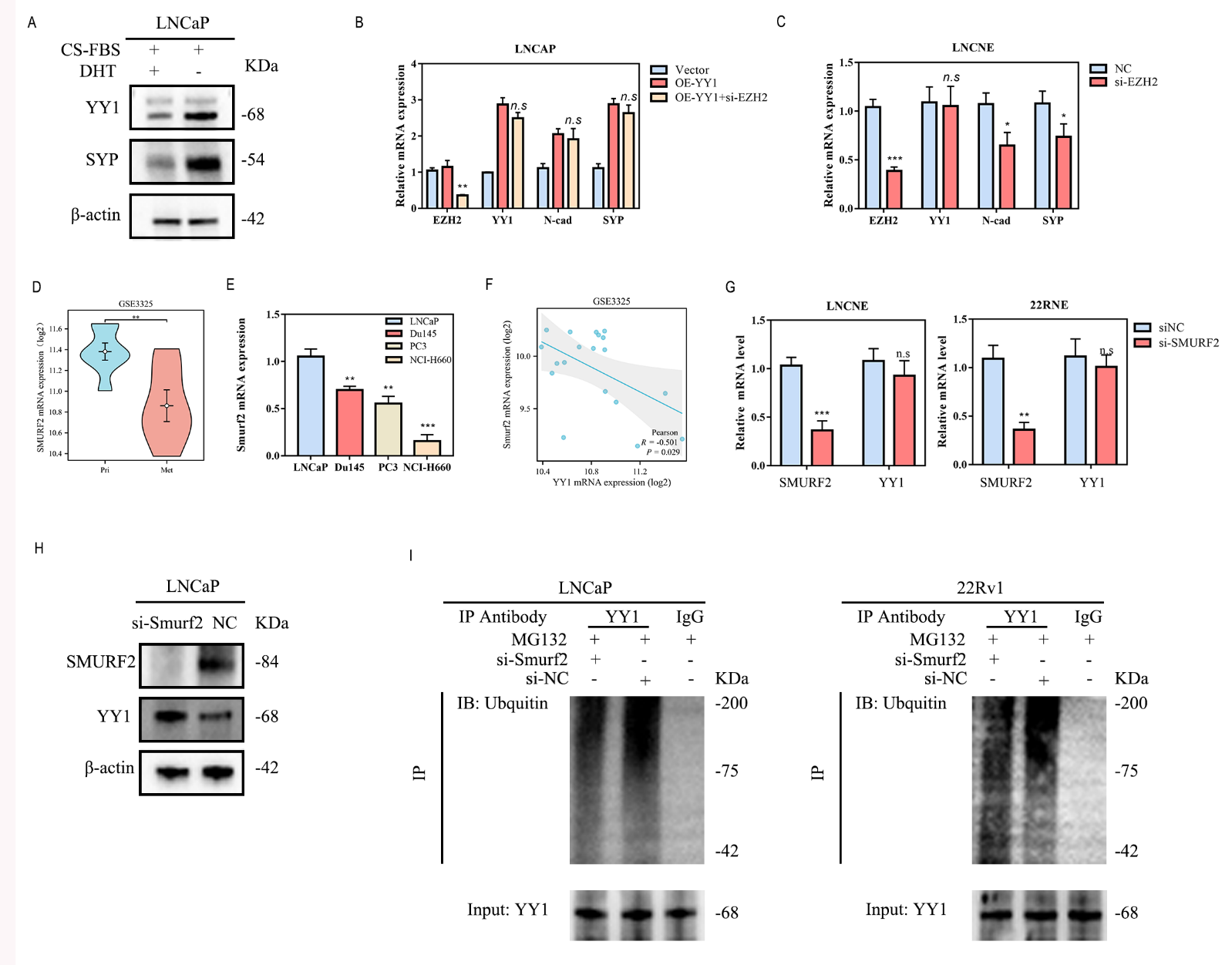


**Figure S5. Upstream regulation of YY1 in NED.** **A.** Western blot analysis was performed to analyze the effect of DHT on YY1 expression after ADT. **B**-**C.** RT-qPCR was used to analyze the regulatory effect of EZH2 on cell plasticity. **D** The level of SMURF2 expression comparing primary PCa and metastatic PCa tissues in GSE3325. **E.** RT-qPCR showed Smurf2 expression in prostate cancer cell lines. **F.** Co-expression analysis of SMURF2 with YY1 using GSE3325. **F-G** RT-qPCR (**G**) and WB (**H**) were performed to analyze the effect of SMURF2 knockdown on YY1 expression. **I.** Western blot analysis was performed to analyze the effect of SMURF2 knockdown on ubiquitination of YY1in LNCaP cell (left panel) and 22Rv1(right panel). n.s, not significant, *p < 0.05, **p < 0.01, ***p < 0.001


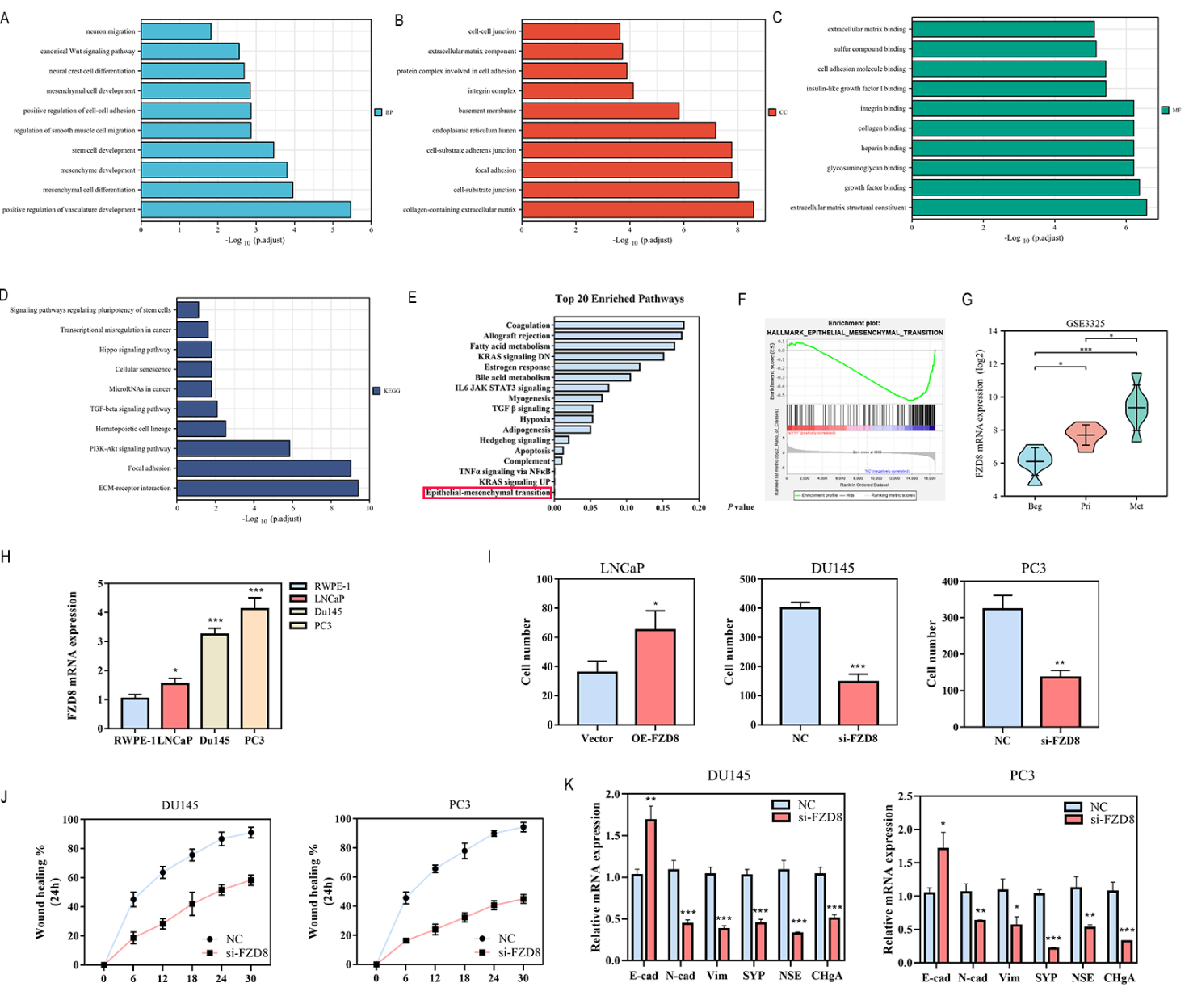


**Figure S6. Functional pathway analysis of YY1 target genes.** **A-C.** GO analysis of YY1 target genes, biological process (**A**), cellular component (**B**), molecular function (**C**). **D.** KEGG analysis of YY1 target genes. **E** The top 20 enriched pathways of YY1 target genes from GSEA. **F.** GSEA showed that the target genes of YY1 were significantly enriched in the EMT pathway. **G.** Violin plot shows FZD8 expression in GSE3325. **H.** RT-qPCR showed the level of FZD8 expression in PCa cells. **I.** Transwell assays showed the effect of overexpression/knockdown FZD8 in PCa cell migration. **J.** Wound healing assays showed the effect of knockdown FZD8 in PCa cell migration. **K.** RT-qPCR showed the effect of FZD8 knockdown in regulation of EMT and NE markers. *p < 0.05, **p < 0.01, ***p < 0.001


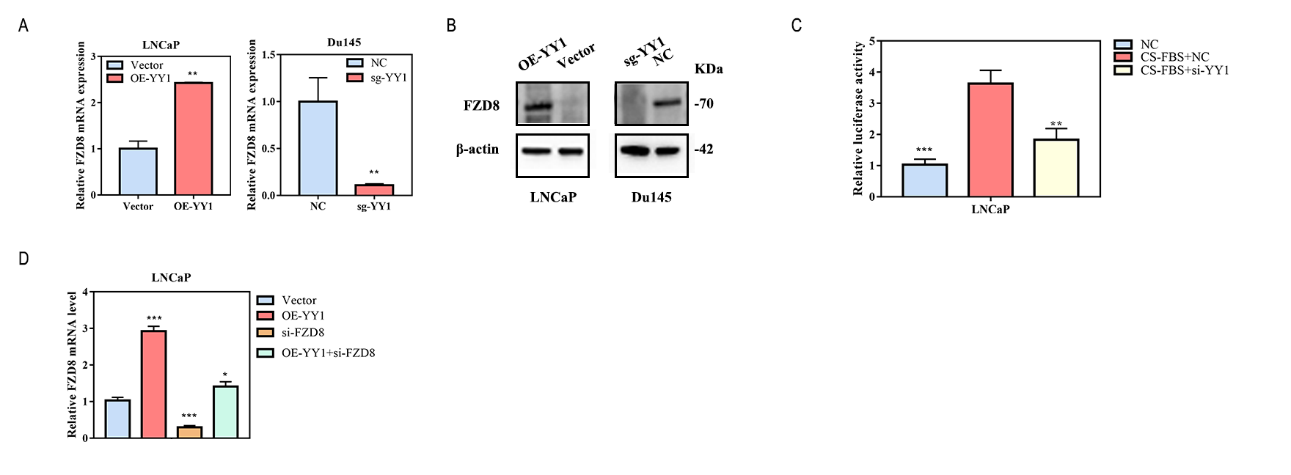


**Figure S7.** FZD8 is regulated by YY1. **A-B.** RT qPCR (**A**) and western blotting (**B**) were used to observe the expression of FZD8 in LNCaP-OE-YY1 and Du145-sg-YY1cells. **C.** Luciferase assays showing the role of CS-FBS with YY1 on the transcriptional activity of the FZD8 promoter. **D.** RT-qPCR demonstrated the transfection efficiency of si-FZD8. *p < 0.05, **p < 0.01, ***p < 0.001


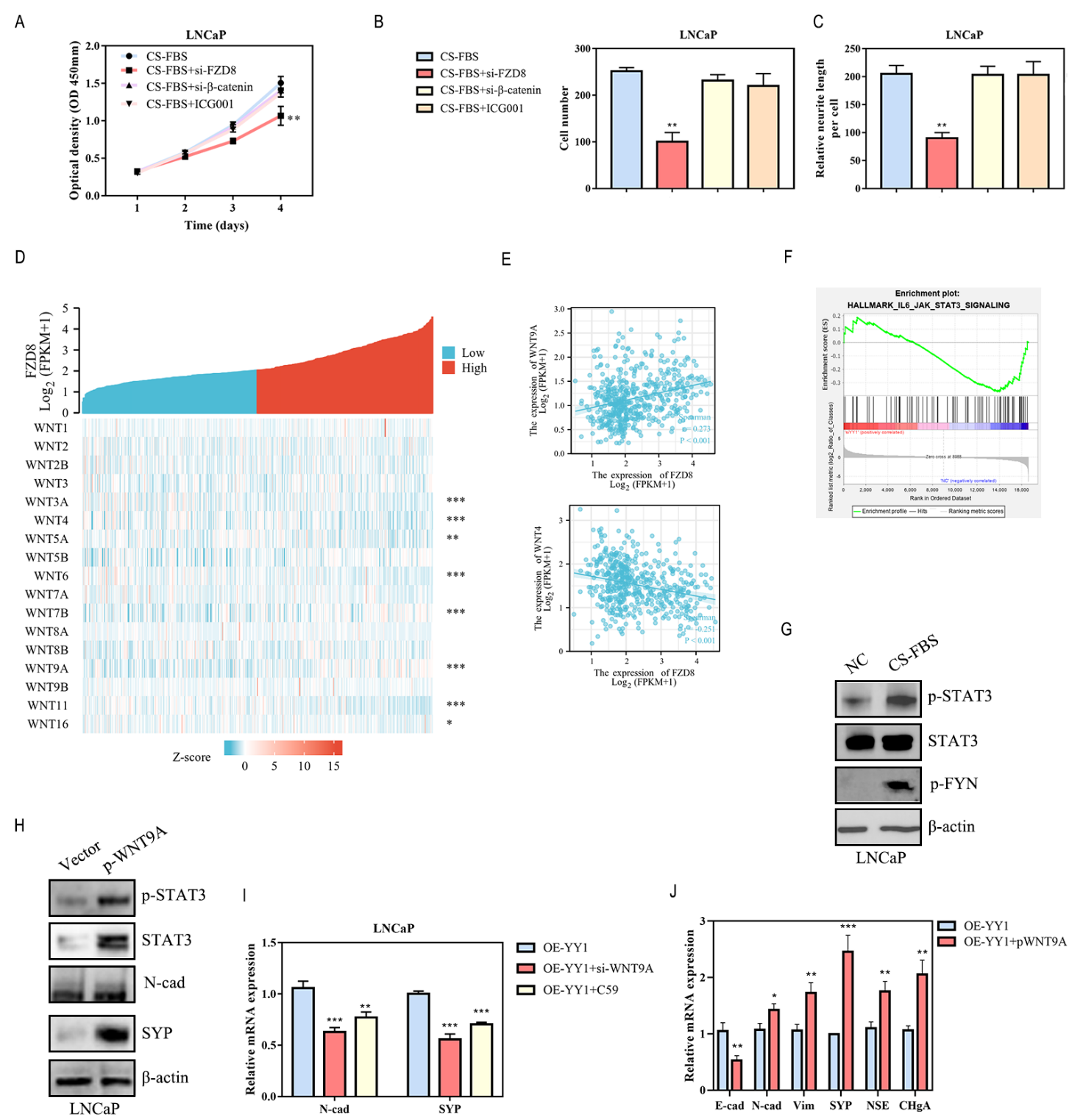


**Figure S8. Wnt9A is involved in YY1 induced prostate cancer** **cell plasticity. A-C.** CCK-8, Transwell, and neurogenesis analyses showing the effect of FZD8 or β-catenin inhibition (ICG001 [2 μM]) on CS-FBS-induced cell proliferation (**A**), migration (**B**), and neurogenesis (**C**). **D.** Co-expression heatmap of FZD8 with Wnt ligands. **E.** Co-expression analysis of FZD8 with Wnt9A (above) or Wnt4 (below). The correlation coefficient was calculated by Spearman analysis. **F.** GSEA showed that the target genes of YY1 are enriched in the IL6 JAK STAT3 signaling pathway. **G.** Western blot analysis showed the effect of CS-FBS treatment in STAT3 activation in LNCaP cells. **H.** Western blot analysis showed the effect of Wnt9A overexpression in cell plasticity and STAT3 activation. **I-J.** RT-qPCR showed the effect of Wnt9A inhibition (**I**) or Wnt9A overexpression (**J**) in YY1 induced cell plasticity. *p < 0.05, **p < 0.01, ***p < 0.001


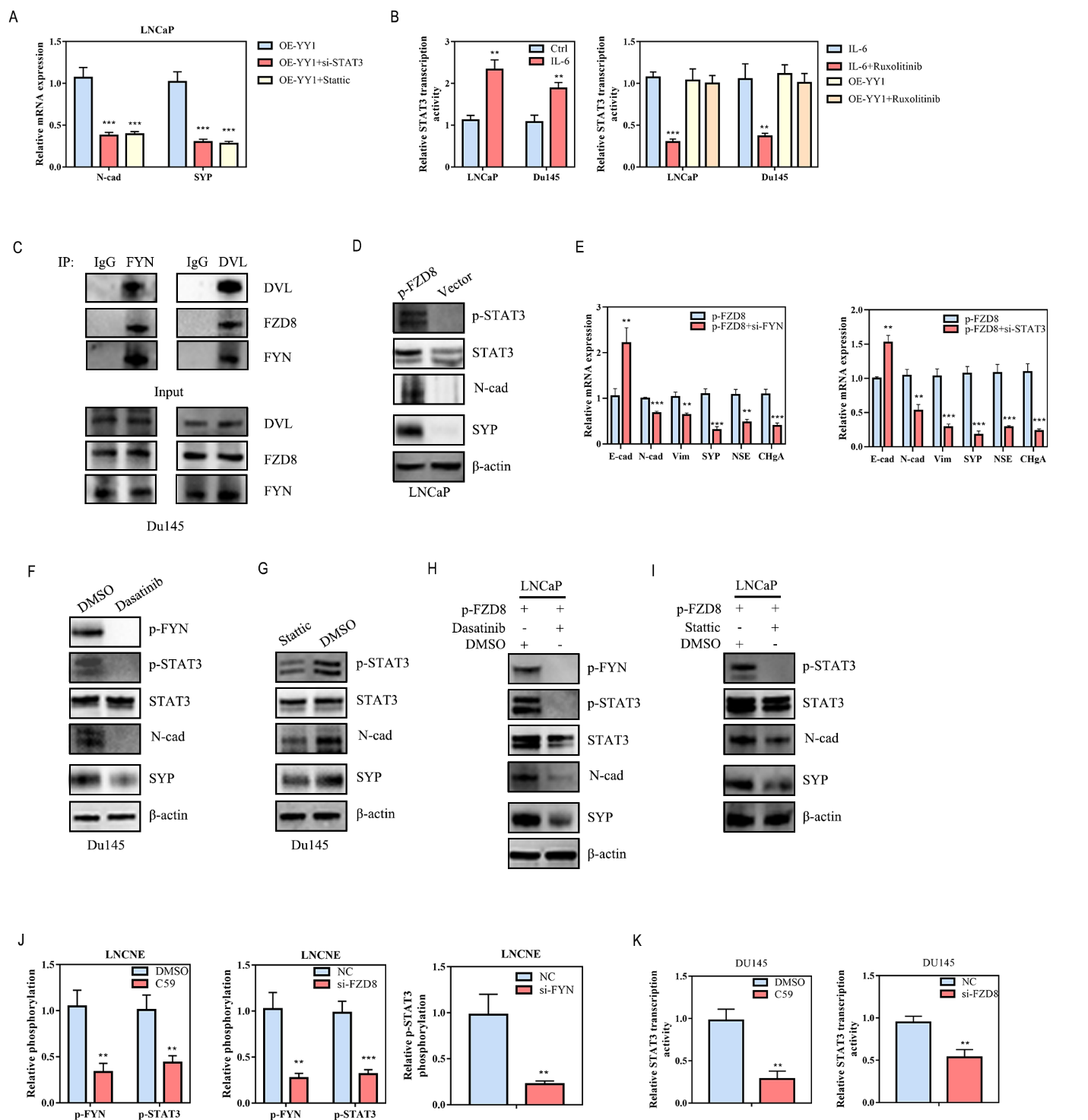


**Figure S9. YY1 regulated** **cell plasticity through the non-canonical Wnt pathway. A.** RT qPCR analyses showing the effect of STAT3 knockdown or STAT3 inhibitors (stattic [10 μM for 24 h]) on N-cad and SYP expression in LNCaP-OE-YY1 cells. **B.** Effect of IL-6 (200 ng/ml for 24 h) on the transcriptional activity of STAT3 in indicated cells (left) and luciferase assays showing the effect of ruxolitinib (3 μM for 48 h) on transcriptional activation of STAT3 induced by IL-6 or overexpression of YY1 (right). **C.** CO-IP showed the interaction of FZD8 with DVL and FYN. **D.** Western blot analysis showed the effect of FZD8 overexpression on cell plasticity and STAT3 activation. **E.** RT-qPCR showed the effect of si-FYN and si-STAT3 on FZD8-induced cell plasticity. **F-I.** WB showed the effect of an FYN inhibitor (dasatinib) and STAT3 inhibitor (stattic) in Du145 or p-FZD8 LNCaP cells. **J.** Effect of C59, si-FZD8, or si-FYN on FYN or STAT3 phosphorylation in LNCNE cells. **K.** Effect of C59 and si-FZD8 on the transcriptional activity of STAT3 in the indicated cells. *p < 0.05, **p < 0.01, ***p < 0.001
