# Supplementary material for: Yin Yang 1 promotes the neuroendocrine differentiation of prostate cancer cells via the non‐canonical WNT pathway (FYN/STAT3)
Source: Clin Transl Med. 2023 Sep 28;13(10):e1422. doi: 10.1002/ctm2.1422 (PMC10539684; doi:10.1002/ctm2.1422)
Supplement: Supplementary file 2 — Table S1. RNA‐seq analysis on YY1 knockout PC3 cells. [file CTM2-13-e1422-s006.docx]

Supplemental Table 1. RNA-seq analysis on YY1 knockout PC3 cells.

| id | foldChange | log2FoldChange | pval | padj |
| --- | --- | --- | --- | --- |
| FZD8 | 9.696756 | 3.277502 | 9.71E-14 | 1.37E-09 |
| KCNMA1 | 9.0405 | 3.176402 | 1.66E-13 | 1.37E-09 |
| IL1RL1 | 8.81459 | 3.139893 | 8.27E-09 | 2.29E-05 |
| CXCL8 | 8.285714 | 3.050626 | 9.58E-06 | 0.005887 |
| FKRP | 7.532468 | 2.913123 | 2.54E-05 | 0.012026 |
| SULF2 | 7.448328 | 2.896917 | 1.87E-07 | 0.000259 |
| TNC | 7.024267 | 2.812348 | 6.02E-10 | 2.50E-06 |
| ITGA6 | 6.234882 | 2.640362 | 5.03E-10 | 2.50E-06 |
| IL1B | 5.711224 | 2.5138 | 6.21E-05 | 0.025136 |
| NOG | 5.566964 | 2.476891 | 0.000135 | 0.043811 |
| ANPEP | 5.393293 | 2.431166 | 2.49E-08 | 5.90E-05 |
| LAMB3 | 5.326531 | 2.413196 | 6.11E-09 | 2.03E-05 |
| LAMC2 | 5.245392 | 2.391051 | 6.18E-08 | 0.000114 |
| PTHLH | 5.071429 | 2.342392 | 2.46E-05 | 0.011986 |
| COL6A3 | 5.011029 | 2.325107 | 1.75E-07 | 0.000259 |
| MACROH2A2 | 4.992376 | 2.319726 | 4.37E-06 | 0.002898 |
| G0S2 | 4.978916 | 2.315832 | 3.88E-07 | 0.000402 |
| NAV3 | 4.961794 | 2.310862 | 0.000133 | 0.043811 |
| YY1 | 4.947127 | 2.306591 | 3.37E-07 | 0.000398 |
| FN1 | 4.840758 | 2.275233 | 3.50E-08 | 7.26E-05 |
| AOX1 | 4.75915 | 2.250704 | 2.67E-06 | 0.00211 |
| ETS1 | 4.649407 | 2.217047 | 4.83E-07 | 0.000471 |
| SORL1 | 4.272321 | 2.09502 | 1.21E-05 | 0.006902 |
| CIZ1 | 4.229619 | 2.080528 | 4.05E-06 | 0.002892 |
| CAV1 | 4.159856 | 2.056534 | 3.60E-07 | 0.000398 |
| SERPINB7 | 4.048701 | 2.017459 | 9.69E-05 | 0.034201 |
| EMP1 | 3.998162 | 1.999337 | 4.19E-06 | 0.002892 |
| KRT19 | 3.859131 | 1.948276 | 2.97E-06 | 0.002241 |
| FAM43A | 3.844912 | 1.942951 | 3.85E-05 | 0.016358 |
| KDR | 3.832143 | 1.938151 | 5.70E-05 | 0.023635 |
| FBN1 | 3.815789 | 1.931982 | 2.76E-05 | 0.012026 |
| SERPINE1 | 3.803448 | 1.927308 | 1.28E-06 | 0.001119 |
| COL13A1 | 3.565619 | 1.834153 | 1.44E-05 | 0.00796 |
| TGM2 | 3.50145 | 1.807953 | 6.78E-05 | 0.026133 |
| COL6A1 | 3.48359 | 1.800575 | 4.57E-06 | 0.002912 |
| NTSR1 | 3.285714 | 1.716207 | 0.000154 | 0.048251 |
| MAP4K4 | 3.235493 | 1.693986 | 2.05E-05 | 0.010965 |
| ADAM19 | 3.163127 | 1.661352 | 0.000161 | 0.049397 |
| PKP1 | 3.143077 | 1.652178 | 2.61E-05 | 0.012026 |
| IGFBP4 | 3.13312 | 1.6476 | 2.72E-05 | 0.012026 |
| PLAT | 3.078881 | 1.622406 | 7.14E-05 | 0.026504 |
| NEFL | 3.032422 | 1.600471 | 0.000112 | 0.038863 |
| ITGA3 | 2.78778 | 1.479117 | 0.000145 | 0.046191 |
| MALAT1 | 0.321015 | -1.63929 | 7.19E-05 | 0.026504 |
| RNA28SN5 | 0.282354 | -1.82443 | 2.64E-06 | 0.00211 |
| RN7SL1 | 0.252115 | -1.98785 | 2.13E-05 | 0.011059 |
| MKX | 0.200461 | -2.31861 | 0.000121 | 0.040855 |
| PLEKHB1 | 0.196429 | -2.34792 | 7.52E-05 | 0.027097 |
| PRR36 | 0.18746 | -2.41535 | 6.53E-05 | 0.025773 |
| SPARC | 0.15198 | -2.71805 | 5.45E-07 | 0.000502 |
| LSR | 0.027992 | -5.15883 | 2.23E-05 | 0.011191 |
| EPPK1 | 0.027019 | -5.2099 | 2.29E-07 | 0.000292 |
| NPTX1 | 0.026221 | -5.25315 | 1.06E-05 | 0.006304 |
| UCA1 | 0.010569 | -6.56408 | 1.66E-07 | 0.000259 |
