# Supplementary material for: Yin Yang 1 promotes the neuroendocrine differentiation of prostate cancer cells via the non‐canonical WNT pathway (FYN/STAT3)
Source: Clin Transl Med. 2023 Sep 28;13(10):e1422. doi: 10.1002/ctm2.1422 (PMC10539684; doi:10.1002/ctm2.1422)
Supplement: Supplementary file 3 — Table S2. Venn results. [file CTM2-13-e1422-s005.docx]

Supplemental Table 2. Venn results.

| Names | total | elements |
| --- | --- | --- |
| Beltran dataset  EMT  sg-YY1 | 1 | FZD8 |
| Beltran dataset  EMT | 5 | SGCB CDH6 LOXL2 GADD45A DPYSL3 |
| Beltran dataset  sg-YY1 | 31 | CALB1 NR2F1 CLDN11 PLEKHB1 C1QL1 B3GALT5 TMCC2 FGF1 KIAA1549L PPP1R37 ZNF711 ARNTL2 TMEM171 DNLZ WNT9A UBE2QL1 C1QL4 LYPD1 JAG2 SIGLEC15 SLC29A4 PLXNA2 MYH15 PCDH20 NFATC2 BPHL SOBP CHN1 DNER ETV1 EPHB2 |
| EMT  sg-YY1 | 36 | CD44 ITGA5 ADAM12 GJA1 TGM2 PTHLH RGS4 OXTR CXCL8 FN1 COL6A2 VIM PTX3 CDH11 ITGA2 FBN1 COL4A2 TNC TPM4 INHBA SDC1 ANPEP ID2 SERPINE1 LAMC2 QSOX1 COL6A3 ITGB3 SNTB1 IGFBP3 IGFBP4 JUN PCOLCE2 SNAI2 PLAUR SPOCK1 |
| Beltran dataset | 1559 | ST8SIA5 RIT2 SLC18A1 FEZF1 AMER2 E2F8 KIF26B RALYL TSPAN11 RXFP3 PDE6A BBOX1 C10orf25 ZNF684 C13orf45 NMBR RTKN2 WASF1 GNG8 CYP11A1 CCNA1 ZPLD1 PBX4 STOX1 C11orf42 CNTD2 C12orf42 SALL1 OTOP2 ADAMTS18 ACOT12 SLC17A1 CFHR5 ZAR1 ARHGAP11A LCORL LDB3 TMEM139 CCDC177 ANGPTL5 FRMD3 OR51V1 GAL3ST3 TPX2 COL22A1 TECTA EPB41L4B RTDR1 OR8B2 B9D1 C2orf73 MEX3B SMIM17 RTN1 KLHL14 PRKCG DEPDC1B LYG1 PROZ DDX11 IGF2BP3 HOXD11 C8A C4orf22 ZBED3 SLC15A5 IZUMO4 NAT8 KCNF1 CCDC28B C16orf92 KCNK3 GPR180 GDA C17orf50 MMS22L C14orf23 MREG CHRM5 SERPINA6 FXYD6 OR10D3 MTNR1B AK7 UNC5A WSCD1 HOXA1 NGF IFI27L1 MYO3B KIAA1009 B3GAT1 KIAA1199 HIST1H2AA CDCP2 MNS1 SOGA2 C15orf41 SYN3 GPR3 GLRA4 JAM3 TAS2R10 OXT KIAA2022 B4GALNT1 TRPM8 GPR83 GPR153 H1FOO CCDC34 RGS16 LRFN5 SLC26A10 CYP2D6 OR4C46 RTL1 GPX7 GINS1 BCO2 POU3F1 CYP2C19 CA14 YBX2 ACSBG2 CIB2 PHACTR3 GPC5 HRK C7orf69 TENM4 ZIC2 C16orf59 C3orf30 AADAC FEV TMEM240 SLC8A1 PRDM9 ISL1 CA11 SLC18A3 GRK1 SPAG11B C1orf61 ANLN KCNA1 SSTR2 C10orf67 BIRC5 TMEM45A C1QL3 WDR54 PLK1 SLC2A13 C1orf101 ANO5 ANKRD6 RTN4R SPIRE2 GALNT13 GBP7 FOXJ1 SCUBE3 LCT AKR1CL1 VWA5A NPPB CNGA3 DRD5 KATNAL1 RIPK4 KNTC1 ANKRD63 DUSP4 FGF17 KCNA2 ANKRD35 AVPR1B HS3ST1 ROR2 FOXM1 SEC16B GRAMD2 CHST1 SLC16A12 GSG2 NPAS1 ETNK2 ANGPT4 CABP1 VAT1L BPIFB2 ZFP37 C12orf40 C7orf33 AMY1B BCL2L10 FRRS1L PTH2 KIAA1211L PLEKHG4 NUP62CL GLRA2 TGFBR3L NEUROG3 MARK1 CDK1 ZNF232 DRAXIN TFCP2L1 STEAP3 FABP5 PAK6 PANX3 TMC2 ASCL5 SLC8A3 OR51J1 C8G FAM163A VAX2 TRIM72 KIF11 SLC24A5 MMP16 IGSF1 CAMTA1 KLHL41 TMEM143 C21orf88 ORC1 LRRC4C TNFRSF11B DRD3 A3GALT2 AIPL1 EXO5 RACGAP1 NAALADL1 TLX1NB SLMO1 CGB5 RAB3A PRAMEF7 SLCO3A1 FAXC CEACAM16 S100A5 KIF18B GABRR2 LCN12 PCK2 HDGFL1 TOX3 CLCN4 DCLK1 MYEF2 DNMT3B ASF1B SLC9C1 DAO SCML2 NDST4 ALG1L F7 ADORA2A PDCL2 RAB41 ZNF774 ADAM23 FOXD3 PDE4C TMEM237 GUCA1B EFHC1 KNSTRN TSPAN7 PPFIA3 C19orf55 COL21A1 GCM2 ADCY5 ZNF334 FOXB1 GPR19 HOXB3 FAM135B PAQR8 CTSV EFCAB7 NR0B1 GRIK2 IGSF11 SMAD9 C3orf52 CDKL4 HTR3A CCDC181 CDC25C CCDC108 ACOT7 RIIAD1 GAGE2B MROH7 LAMB4 STRIP2 ECE2 FAM24B PALM2 AGT ARHGAP33 C1orf111 NACAD CGA SCD5 LRRC10 IGFL4 PSPN ACTRT1 TMEM44 PNMAL2 DBH NANOS3 SAPCD2 CACNA1B DEFB105B SKOR2 DMPK OR8A1 SLC10A4 C9orf173 AKNAD1 MUTYH CAPN9 NEBL FAM71E2 CALCA CEL ANKS6 PLCXD2 DLL1 RGAG4 C19orf45 CACNG4 HLF ENHO NPHS1 TPBG SPRY2 YIPF7 RET PKIB COLEC11 ZP1 HOXD13 HOXD4 IGDCC3 TRIM55 SLC9A3 C2orf81 SNX10 SLC39A5 VSIG8 TLE2 CEP85 MAMSTR MTTP TSHZ2 AURKA KIAA1755 OR8D1 GPRC5C KHK ESRRG KIF4B ECM1 GIPR ZNF540 EFNB3 C1orf51 AKR7A3 HOXA3 KISS1R TEKT3 MAGEA9 AFP TIAM1 KIF14 PTGER1 SCN11A ZCCHC18 IL17C ETV2 IL17RE C17orf53 GPR12 LRRC53 MYLK2 MAD2L1 CUZD1 PTPRH KCNA4 NMNAT2 PCLO SAMD7 TPBGL C4orf48 DLX6 CCDC81 SCNN1D TMEM246 UNC5CL SLC32A1 LEFTY1 SEBOX CCL27 SCN1A CLPSL2 BHLHE22 STRC MYBPH AURKB HAPLN4 CPA2 CDHR1 C2orf78 LRRC66 NBPF6 SV2B FAM149A RIC3 MAP10 LRTM1 CDKN2D PPARGC1A RCOR2 KCNK10 RNF182 TENM1 FSTL5 LCN1 RALGPS1 C20orf173 CLDN9 ZDHHC11B SLC17A9 PITX2 CCM2L C17orf100 HMX2 C2CD4C G6PC2 LCN15 HOXB8 CHRNB1 ANKS1B LIN28B MFSD2A CDH18 GATS P2RX6 CNTF C2CD4B FA2H SGOL1 TTLL7 SPOCK3 MEIS1 ANO9 GAS2L3 ATP2B2 JAKMIP1 GGTLC3 UNC79 FBLL1 BEX2 FAM3B BLM KCND3 CHST4 RIMS3 SYCE3 BVES TMEM253 CFHR4 ELAVL2 ABCC2 PRMT8 KIF4A ADRA1B RAB9B CADPS SNCAIP NCAPG2 PHOX2A SSMEM1 LNP1 RGL3 ZNF726 KCNK17 MAGIX ITPKA NGB AOC1 BRSK1 CLUL1 LZTS1 CPN1 KIT PECR RHBDL3 B4GALT6 WFIKKN1 HSPA2 EPHA7 THPO MS4A8 MORN3 NPM2 FAM229B ZNF439 LGR5 NKX2-8 PTGES3L NT5DC4 RAB33A DBX1 STK32A PANK1 TFAP2D ZFP30 AK9 MYCL ROBO1 TCP11X2 LRRIQ3 HOXC5 BARD1 SMCO2 HS3ST6 ICA1L FAM131C GRIA1 TPH1 FAM19A1 KCNC3 CA9 C19orf47 TLX1 RGS8 SPDL1 SLC36A4 ZNF296 LGSN MPP2 INPP1 PTCHD2 NPAS4 USH2A CFHR1 KIF2C C6orf141 NRAP SLC25A34 NEGR1 SLC7A4 DMRTA1 MAP1A HOXA5 FBXO5 FGF12 INPP5B PAK3 EFCAB5 GCM1 CHN2 H2BFWT LRRC34 CYP2C9 AGPAT4 TTLL2 UTS2R ACVR1C GCGR GALNT12 SALL2 CA5A IGFBP2 EIF4E1B LRFN2 USP11 C12orf39 MEIOB SLC17A4 TAS2R1 TAL2 TOX C5orf34 TRPC1 SPAG11A STK33 KLF12 SLCO1B1 SST SLCO1A2 NR6A1 RUNDC3B NEUROD1 SGCZ CENPA DIRAS2 TPPP3 HAPLN2 CALB2 OTP IFNA5 TMOD2 CDCA7 JPH4 SLC44A5 IGFN1 DENND2A ADAMTSL2 USP2 KIAA1024L SLC5A9 ABCG4 MEGF11 SDK2 BAIAP3 MELK DPY19L2 DENND5B C1QTNF2 PLCD4 KLC3 SSUH2 FOXN4 OVOL2 ESRRB CCNJL ZNF599 KCNMB3 FAM170B ASPHD1 CAMK1G CPNE9 KLK12 HCG27 CDC20 CCKBR OTOF DOK6 HS3ST5 IL36G DRP2 DFNB31 OSTN RPH3AL MAP6 RETNLB PPP1R14D SLC6A20 DNAJC12 CCDC40 SLC16A11 DRGX DNAJC18 SLC24A2 MANEAL PER3 NDC80 PITPNM2 ZNF653 SHISA8 L3MBTL4 PPM1E GCHFR ZNF780B CDKL2 KANK4 OR1L8 NAGS ZNF713 SPERT QPCT LRRC10B C1orf185 OSBPL6 MFI2 CCDC70 KCNK12 GRB14 FOXE3 ULBP1 PLK4 LMX1A KIAA1841 RFC4 OIP5 ACAA2 RXFP1 DCAF8L1 DCDC2 LCE1E HAUS8 MAPK8IP2 SULT4A1 GTSE1 SHC3 POU3F2 LRRC73 FIGN MIA SP9 SCGB2A1 CCDC151 NUTM2F LIN7B HOXA7 BUB1 LINC00908 HOXD1 KIAA0087 GBX2 SPAG4 NUF2 PBK COCH GAB2 SLITRK6 C1orf220 PCYT1B ADPRHL1 FOXD4L4 HUNK C5orf54 PRB3 BMPER CBLN2 MSANTD3-TMEFF1 DUSP10 PPP1R17 CSAG1 PRR11 APOM SPATA16 SKA1 B9D2 DEFB106B FOXA3 NEB SCGB2B2 ASGR1 C18orf56 TROAP FGF14 EPHB1 FBXL2 SSBP2 ELOVL4 OR8B12 ADAMTS6 PRICKLE2 CCL24 CDCA5 CSRNP3 HABP2 LPHN3 DLL4 UBE2T FBXO15 NANOS2 HSD11B1L ATP2A3 FAM151A ZIK1 DMRTA2 EPOR ADCY1 PLXND1 TMPRSS15 SLCO6A1 SUV420H2 OR51M1 GJC3 CCDC129 ISYNA1 C4orf50 TMEM89 OR8G5 RAB39B C2orf48 SHF ZFR2 ASB15 FOXG1 CACNA2D3 NME9 AJAP1 PROC HSD17B13 C7orf13 CACNA2D2 ECT2 SLC25A42 LYPD6 SLC4A10 PCDHB6 DEPDC1 KIF23 B3GALT2 ZNF165 CCDC74A C1orf95 VWC2L BEST3 CABP7 ZNF618 HFM1 XCL1 CEP57L1 HORMAD2 KLHDC8A BPIFB4 MCF2L DACH2 SPEG CLNK TUBE1 DSPP SYNPO2L DACT2 SKA3 NCAPH SHD DNAJB13 MYBL1 SHC2 MST1R GFRA3 IRAK1BP1 MFSD6L EGR4 LETM2 DIRAS3 VIL1 KIAA1324L MDGA1 CACNA1D C1QTNF4 KCNH3 H2BFM FAM183A VCX3B DUSP19 ASB4 LMO7 PAM TMEM178B BTBD3 PRDM8 SYT1 GOLGA7B ACRV1 HOXD8 KRTAP12-1 PAGE1 GRM3 GDPD4 SEMA5B FUT1 CCDC74B SMC2 COLCA2 TMEM255B NPHP4 SFRP5 DLG2 EFNA5 MTFR2 RCCD1 TUBB2A ZIC5 ASPM FITM1 UBE2S CKMT1B TSPAN12 VWDE CNTLN ATP5SL PTPRD OCIAD2 KLHDC7A PPP5D1 STXBP5L MAGEA2B TMSB15A EBF4 PRG4 HRH3 CEND1 PTPRN2 MAOB MPZ C11orf52 TMSB15B ABCA10 PLA2G12B BPIFA1 HDX HS3ST3B1 IGSF10 MMD2 ALLC RBP4 CT47B1 CEP78 CDCA3 TCP10L PNMA2 ZNF491 GPR137C CES3 TTC36 RTN2 CNIH2 ZNF705D HSD17B3 CORT PAK7 KCNS2 CHD7 CHRNA1 HOXA2 SNPH GSX2 IAPP HOXD12 C10orf91 ID4 RXFP4 SOX5 CDH22 KIF6 TAS2R42 DSCAM BRIP1 INA SLC6A1 CALCB GNAZ PAX6 CDON RTP2 POU4F3 IL37 NBPF4 IL17REL THSD7A OVCH1-AS1 UTS2 SLC22A7 GNAI1 TAT GOLGA6B TMPRSS5 STIL ADAM28 NRL FFAR4 C11orf94 RASGEF1B ACOT11 PGAP1 HOXD10 ODF3L1 PIH1D2 TEX35 SBK3 DZIP1 KIF17 MAG COLEC10 EFHB LBX2 RINL CCNB2 DLK2 RAB3B SEMA6C GRIK5 POLE2 FGF9 ARHGAP19-SLIT1 TMEM196 PRC1 SLC16A13 CSMD2 CYP2C18 PDX1 ARHGEF28 GSX1 IGFBP1 GRIN2D CASC5 CEACAM5 GGT7 GPR152 ASPDH FUT9 MYT1L ZNF878 PMP2 TBR1 SLC4A9 TDRD12 TMIGD1 ROPN1B CDT1 PRSS50 LRRC49 SCN2A ALDH8A1 ASIC2 SLITRK3 CHRDL2 ZNF300 CENPW MASP2 POU6F2 RIBC2 SLC7A9 QRICH2 CEP55 LINGO2 KIAA1377 F13B MEOX2 STK31 GRM5 KIRREL2 AMH KCNG3 MTMR7 CCDC33 MGAT4C OR52B1P TTC34 PCDHB10 DEFB105A SPACA4 ULBP2 CA10 CEP76 RRM2 PLEKHG4B CLPSL1 VCX3A TOP2A CCDC38 WDR72 UNC93A SPTB PLCZ1 NRGN SCN1B A1BG GIF PPP1R36 CKMT1A LPCAT4 LOH12CR2 ALDH1A1 NMNAT3 TMEM35 FMO5 C19orf77 HELLS FANCI SH2D7 PNMAL1 DBF4B C19orf73 DLX5 RAD54L ITIH2 CAPS2 ZNF367 CNGA4 TMEM38B CDC45 CACNA1S KIAA1456 DNAJC28 FOXS1 MMP26 PCDHB16 ZNF483 CCDC136 SEMA6D KCNJ3 MYCN IL22RA1 AGRP PCDH8 RPS6KL1 RGS22 ERO1LB NTF3 ABCB4 SMOC1 TCEAL5 NKX6-2 EPM2A LDHC KRTAP5-3 SPC25 SFTPB CAPZA3 CNTNAP2 AMDHD1 ZNF705G ST6GAL2 RAX CDK5R1 GDF11 OR8D2 PDE6B TERT NEUROG2 GABRR1 USP41 RYR2 BEX1 OXCT2 ZNF705B ZDHHC13 HOXA4 KCNK13 PTCHD4 FEZF2 SLC26A9 SSX2B LY6H STRA6 KIF18A MAP2 SPA17 ZNF853 TNFRSF11A CXorf27 PROM1 C1orf222 ZNF569 FGF5 CHIA BARX1 CEACAM7 ASB18 LYPD5 PTPRN WFDC2 DYNC1I1 HIST1H2BA C18orf42 GRIK3 NPSR1 IFNLR1 HOXA9 KRTAP6-1 INHBE DCPS TOX2 FBXW7 MRGPRE OR11G2 ABCG5 UBE3D SERTM1 LIN9 BUB1B FAM163B CCDC173 SPATA6L SOX1 COX6B2 PRPH KCNH5 GOLGA6C FGF10 MAGEA1 NRIP3 NOS1AP GPC2 WDR76 DGCR6 DLGAP5 KCTD19 TMEM17 SKIDA1 LRIT2 HJURP FABP6 TSPEAR ZSCAN31 TCERG1L TMEM108 TMEM198 CLYBL KIAA1614 KCNC4 KIF24 ZSWIM5 VN1R1 RGS9 SYNPR CCDC168 KRTAP4-1 C1orf226 CFHR2 TMEM163 C5orf60 CLVS1 ADAMTS13 RGAG1 BFSP1 HAUS5 LRRC20 FLRT3 CA8 MB21D2 CKAP2L SEZ6L FBLN7 LMO3 GRM7 CYP2C8 CECR6 GPR142 ARC NHS PCDHB12 C19orf68 C9orf24 TCP11X1 LHFPL5 C2orf70 SPP2 CLSPN DTL OFCC1 GNA14 RNF186 ATP6V1G2 RLTPR KIAA1024 CDH7 PCDHB1 RBMXL2 ACMSD CLIP3 LOXL4 C15orf56 EFCAB2 NCALD METTL4 BEST2 TTC23L CDCA2 SLITRK1 ILDR2 MSLNL RIPPLY1 NMUR2 SPSB4 PEMT C10orf111 SARDH SOHLH1 DNAH3 CPA5 C9orf53 HMMR PPP2R2B KAAG1 UNC5D KIAA0319 TMEM59L KIRREL3 HEY2 CHRNA3 C8orf56 CHAF1B SCNN1A NRCAM RADIL KCNB1 C1orf137 EXO1 HMX3 PDZK1 PCP4 FCN3 NRXN3 RIMS2 GRP C12orf56 ZNF488 KRTAP10-10 PKDCC FRMPD1 CATSPER2 CUEDC1 TMEFF1 CENPI STAMBPL1 UCN3 STARD9 FABP12 MS4A12 GPRIN3 STAC CRTAC1 CXXC4 KRTAP5-5 NOVA1 C9orf40 SPRY4 NMU KCNH7 CDH19 AURKC SOWAHA TP53TG3D TAAR1 PITX3 AHSG EYA4 RND2 KIF20A PRAMEF10 SSX2IP LRP4 KSR2 METTL11B RGS17 WFDC10B ASTN1 MLXIPL C2orf50 KCNMB2 SGOL2 ATRNL1 CENPK WFDC8 FOXD4L5 CHRNA9 TUBB8 SOX11 RBP3 CEP152 ARSE ZMAT4 SHC4 WRAP53 TRIM46 SYCE2 HPSE ZNF19 SLCO5A1 C21orf90 ATP8A2 TIGD3 FGF21 MT1H BSND SCRT1 HOXC8 PPP1R14C C1orf127 PRDM15 FOXD1 POMC WNT4 DEFB103B FAM181A F12 FAM57B NCAN GKAP1 SHOX2 HOXA6 FANCE CRTC1 ZNF662 AGBL2 FREM1 ADAM22 HAMP LMO1 TMEM132D PCDHB5 NPS DLK1 HOXD9 UCP3 CCDC89 KIF21B LRRIQ4 EFCAB8 APOA1 NXF5 TAS2R38 MYLK3 MYOZ1 SERP2 TTLL8 NPBWR2 FAM111B PEX5L FUT2 TAAR8 MCF2L2 CDKN2A C18orf54 LIN28A TTLL10 CCDC67 NKX3-2 OR51B5 CIB4 ABCC8 MACROD2 TBX6 EPHA8 ACOX2 POPDC3 OR8B3 TM6SF2 HOXB7 TMTC1 EPHA10 STMN2 GPR111 HCRT B3GALT1 HGFAC RGS7BP C4orf26 CBY3 MMP10 ATOH7 NRG1 TTK CA6 FSD1L ESPN ARHGAP40 PPM1N SLC4A11 FAM81A UGT8 MYH7B PLEKHA6 HOXB2 MYO18B R3HDML RUNDC3A CDKN3 CCDC178 GAD1 FAM132B GPR39 DNAI2 ADAMTSL1 NCAPG KLHL3 TFF3 CCDC148 TMEM88B FAM222A DPP10 NTN3 PAX5 ATP6V1B1 TEX101 MSANTD4 MCIDAS DTNA SNAP91 PPP4R4 AMIGO2 SLC17A8 C21orf58 CYP24A1 GLOD5 FXYD6-FXYD2 NPAS3 NPFFR1 IFI27L2 COL6A6 LCN8 PIPOX KHDC1 ADD2 RNF183 C2orf74 C2orf66 MYBPHL NEK2 FMN2 SLC38A11 GKN2 NPTXR SCN9A SLC5A8 SOX2 GINS2 GRIA2 WDR66 ELMOD1 VCX2 SPC24 CENPF NUSAP1 BMP8B OVOL3 USP27X SLC6A17 HHATL PAH FAM171A2 C2CD4A NYAP2 TCP10L2 ARTN N4BP3 BEAN1 RDM1 RFX2 C3orf67 GNB3 CCDC88A GHRH SLC2A11 TRIM50 ARNT2 SOAT2 GPLD1 RPL39L LIPT2 HMSD KCTD14 TP53TG5 XRCC2 APBA1 |
| EMT | 51 | FSTL1 LAMA2 PCOLCE COL1A1 FBN2 LGALS1 FAS LOXL1 CCN2 ITGB1 VEGFC SDC4 WNT5A NT5E CRLF1 AREG GREM1 PLOD2 TNFAIP3 LAMC1 IL6 FERMT2 ECM2 DAB2 CD59 SERPINE2 SCG2 MMP14 MYLK PDLIM4 EMP3 THBS2 CXCL1 HTRA1 COL16A1 CADM1 COL4A1 MATN2 MEST FSTL3 CXCL6 WIPF1 COL5A1 PMEPA1 SLIT2 PMP22 GEM TFPI2 TGFBI COL12A1 PVR |
| sg-YY1 | 318 | KCNMA1 VAMP2 PFKP SLC10A7 PLAU GALNT3 CTNND2 C1R NTSR1 ZNF385D ID3 SERPINB5 IQCK BIVM-ERCC5 RFX5 PKP1 FGFR1 SORL1 ECH1 ITGA3 EFNB1 CLDN4 SSX6P GLT8D2 CD55 SHISA3 ZBTB22 ST3GAL1 RASL10B DPP4 MUC3A DMTN GTF2IP4 HBEGF RIN2 LAMP3 FAM86B1 TNXB VIM-AS1 ADAM19 FKRP GPCPD1 TMEM200A KCNH2 PLXNB1 RNF145 LTBP1 NRP2 ARL4C VASH1 SERPINB7 LPXN PCCB IL1RL1 FAM86DP RBM38 PCDHA11 PIM1 SH3PXD2A PIK3R3 KLF4 FAM83H ZNF805 ARHGEF16 EMP1 YY1 OXCT1 NPHP3-ACAD11 ALG6 LAMB3 ACOT2 AP2M1 HOXA10-AS PRR36 RNA28SN5 LIMA1 FOXL1 PRKAB2 ANO1 NUDT4P2 CADPS2 FRMD6 TUBA1A PDGFB ABCC3 GDAP1 CNTNAP3B UCA1 HR SYNJ2 SUMF1 LHFPL6 NPR3 CPM NEFL GALC CDC42EP2 P2RY2 XXYLT1 HAS2 ITGA6 BAG3 SULF2 SEMA7A USP43 SCAMP5 SELENOM TNFRSF19 PLCXD3 KLHL4 PODXL H1-10 RBP7 CAND2 PRSS3 CDKN2B FUT8-AS1 G0S2 FAM86C1 SETBP1 AIF1L TCEAL4 ITPR2 CHST2 SEMA4B TXNDC16 ACHE CTSF POU2F2 MBD2 SERPIND1 FUT8 PKP2 COL13A1 LPAR2 GALNT10 PSME1 GLMP POGLUT1 CHMP4C HMGA2 CD109 BCL2A1 TOR1A PCSK1N MOCS1 ADGRG1 KIAA0040 ARHGDIB NPAS2 ARSI FAM111A PDCD6 KDR C10orf55 HAS3 GIT1 C1S EPPK1 MAN1A1 DPF3 FLT1 GPR65 TMEM158 TSPAN15 LATS2 NIBAN2 MGLL ITGA2B STX1A ACY1 NGEF NAP1L3 MFGE8 IFI6 SH3TC2 SLC36A1 PLAAT3 ADAMTS1 SMARCD2 PLAT ANOS1 ITGBL1 KLHL15 NDRG1 LRATD2 ADAMTS15 CAPN2 ERBB3 IGFBP6 CLDN14 WNT7A TP53INP1 CCDC80 RHOB TET2 ENDOD1 CNTNAP3 GLIS3 MDK SLC20A1 ADGRL3 ICAM1 CAV1 RN7SL1 TNFRSF21 ISM2 SLC6A8 CELSR1 ETS1 PIGS NID2 PLA2G7 TMEM200C MCC FAM43A CHCHD10 TNRC6C FSIP2 HEY1 NTNG1 GNG11 EIF4E3 BRINP2 PRDX2 TFPI SMTN COL4A6 LINC00888 CYB5R2 KRT19 BSCL2 TNFRSF18 ZNF185 NAV3 TM4SF1 OSBP2 MX1 INSR PLPP3 TGFBR2 CIZ1 MYL9 GACAT2 FERMT1 CDKN1A NOG LSR SLC7A1 CORO7 CAB39L NPTX1 RASSF8 USP12 CIRBP EPHB6 FAM107A CDCP1 CCBE1 SHISA2 ATG12 MALAT1 ENC1 TNFRSF6B CCN3 IL1B NEURL1B GABBR2 ABLIM3 COL6A1 ARHGEF25 RASL11A MKX WASH3P SPARC CTSH MAP4K4 ZNF385A SYNPO THSD4 HES4 SEMA6B FBXO6 CLTB FGF7P6 AHNAK2 TMSB4X FAM20C IFITM1 BCYRN1 NOTCH3 MLPH MYO1D TRNP1 CYFIP2 ITPRIP FJX1 SELENOT AOX1 FBXO2 FKBP1A FOSL1 PRKCA RN7SL2 GPAT3 SEMA3C MACROH2A2 NDUFA5 SAV1 AS3MT FRMD4B KLHL24 DUSP8 STEAP1 DHRS2 PCIF1 DARS2 RNF169 |
