# Supplementary material for: Yin Yang 1 promotes the neuroendocrine differentiation of prostate cancer cells via the non‐canonical WNT pathway (FYN/STAT3)
Source: Clin Transl Med. 2023 Sep 28;13(10):e1422. doi: 10.1002/ctm2.1422 (PMC10539684; doi:10.1002/ctm2.1422)
Supplement: Supplementary file 4 — Table S3. The information of primers, siRNAs, overexpression/knockdown lentivirus. [file CTM2-13-e1422-s002.docx]

Supplemental Table 3. The information of primers, siRNAs, overexpression/knockdown lentivirus.

The primers used for PCR.

| **Primers (5’ to 3’)** |  |  |
| --- | --- | --- |
| **YY1** | Forward | GCCCTCATAAAGGCTGCACAAAGAT |
|  | Reverse | GTGCGCAAATTGAAGTCCAGTGAA |
| **FZD8** | Forward | CTACATGCCCAATCAGTTCAAC |
|  | Reverse | TGCACAGGAAGAACTTGAGATC |
| **GAPDH** | Forward | GGAGCGAGATCCCTCCAAAAT |
|  | Reverse | GGCTGTTGTCATACTTCTCATGG |
| **Vimentin** | Forward | AGTCCACTGAGTACCGGAGAC |
|  | Reverse | CATTTCACGCATCTGGCGTTC |
| **E-cadherin** | Forward | GAAGCTGGCTGACATGTACG |
|  | Reverse | CTCAAGGGAAGGGAGCTGAA |
| **N-cadherin** | Forward | CAGTCTTACCGAAGGATGTGC |
|  | Reverse | ATCAGCTCTCGATCCAGAGG |
| **SYP** | Forward | TTAGTTGGGGACTACTCCTCG |
|  | Reverse | GGCCCTTTGTTATTCTCTCGGTA |
| **NSE** | Forward | CCGGGAACTCAGACCTCATC |
|  | Reverse | CTCTGCACCTAGTCGCATGG |
| **CHgA** | Forward | GCGGTGGAAGAGCCATCAT |
|  | Reverse | TCTGTGGCTTCACCACTTTTCTC |
| **WNT1** | Forward | ACAAAATTGTTACTGGGACG |
|  | Reverse | ATGTGCTTACTCATTAGGGC |
| **WNT2** | Forward | GTGACAGCCTCTCTCCGCAG |
|  | Reverse | TAGTCACCAGCCACACCTCG |
| **WNT2B** | Forward | TGGATGCCAAGGAGAAGAGG |
|  | Reverse | GTACAGGAACCACTCACGCC |
| **WNT3** | Forward | ACGAGAACTCCCCCAACTTT |
|  | Reverse | AGATGCAGTGGCATTTTTCC |
| **WNT3A** | Forward | ACCATGTTCGGGACCTATTCCA |
|  | Reverse | GCCTGTAGCATCTCGCTTCCA |
| **WNT4** | Forward | TTCAGGAGCATTGGGGAC |
|  | Reverse | TGGCGACTCTTCTAGGTAGAC |
| **WNT5A** | Forward | ATTCTTGGTGGTCGCTAGGT |
|  | Reverse | TGTACTGCATGTGGTCCTGA |
| **WNT5B** | Forward | CTACTTGGACGAATCTCCCGACTAC |
|  | Reverse | CAGCATAAGAGTCCACAGCCATCC |
| **WNT6** | Forward | ACAACGTGGGATTCGGCTAC |
|  | Reverse | TCCCGACCTCGTTGTTGTG |
| **WNT7A** | Forward | CCTTGTTGCGCTTGTTCTCC |
|  | Reverse | GGCGGGGCAATCCACATAG |
| **WNT7B** | Forward | TTCGGCCGCTGGAACTGCTC |
|  | Reverse | TGGCCCACCTCGCGGAACTTAG |
| **WNT8A** | Forward | TGTTCTCCTGGCTTGTGG |
|  | Reverse | CTGGGTGGAACGGTATCTG |
| **WNT8B** | Forward | ACTGGTCCAAAGGCTTACCTG |
|  | Reverse | GGCTCTCTCAGGGCAGTTC |
| **WNT9A** | Forward | TCGAGTGCCAGTTCCAGTTC |
|  | Reverse | AAGGCAGTCTCCTTGAAGCC |
| **WNT9B** | Forward | CTGGTGCTCACCTGAAGCAG |
|  | Reverse | CCGTCTCCTTAAAGCCTCTCTG |
| **WNT11** | Forward | CAGCGTGTCATTTTTTTACC |
|  | Reverse | TGTTCACAGAGAGTCCCATTC |
| **WNT16** | Forward | GCCCCGTTGTGCCCAATATACC |
|  | Reverse | GACCACGGCGTAAAGGGAAGAC |
| **MCL1** | Forward | GGGCAGGATTGTGACTCTCATT |
|  | Reverse | GATGCAGCTTTCTTGGTTTATGG |
| **CSF1** | Forward | AGACCTCGTGCCAAATTACATT |
|  | Reverse | AGGTGTCTCATAGAAAGTTCGGA |
| **SOCS1** | Forward | CTGCGGCTTCTATTGGGGAC |
|  | Reverse | AAAAGGCAGTCGAAGGTCTCG |
| **PLAU** | Forward | GCTTGTCCAAGAGTGCATGGT |
|  | Reverse | CAGGGCTGGTTCTCGATGG |

The primers used for ChIP assay.

| **FZD8-ChIP-primer** | **Sequence (5’to 3’)** |
| --- | --- |
| F1-Up | GAACGCATTGCTGAGGTGTAG |
| F1-Down | TCCATTGAAGAGCGCCTGCT |
| F2-Up | AGCAGAAGGCTGTTGAATTAGC |
| F2-Down | GGAATGATGTGCTGAGCCACT |
| F3-Up | TCTTTCCCTGCTGCCTATCTC |
| F3-Down | AGTCTTCCTCTCTCGCGCT |
| F4-Up | TTTCCCGGCATTGTTAGCCA |
| F4-Down | ATTATGTGCGCAGACCACCT |
| F5-Up | TGCCGGTGCCTAAATTCTATTG |
| F5-Down | GCAGCTTTTACTTCCGGTGC |
| F6-Up | AAAAGATAGCCCCAGGTCCC |
| F6-Down | ATGCCCCATCATTAGCAGGG |
| F7-Up | ACGCTACTTTCTTGGCCGAT |
| F7-Down | GCATGATAGCTAAAGCAGGGG |
| F8-Up | TGGCCTACATCTAACCCACG |
| F8-Down | GACTCTGGGATCTGCTCTCAA |
| F9-Up | GCATGTTGGCTTTCTCATGTAGC |
| F9-Down | AAGCTGGACATTTTCCCACTGT |
| F10-Up | AGACCTGCATCTGTCATCACT |
| F10-Down | AAGGCAAAGAACCAGCACTT |

| siRNAs or sgRNA (5’ to 3’) | |
| --- | --- |
| sg-YY1 RNA | GATGTAGAGGGTGTCGCCCG |
| si-β-catenin | UGGUUGCCUUGCUCA ACAA |
| si-FZD8 | GGCUACAACUACACCUACATT |
| si-STAT3 | GCAGCAGCTGAACAACATG |
| si-FYN | GGAUAAAGAAGCAGCGAAA |

| YY1 overexpression lentivirus sequence |
| --- |
| GGCCGTTTTTGGCTTTTTTGTTAGACGAAGCTTGGGCTGCAGGTCGACTCTAGAGGATCCCGCCACC**ATGGCCTCGGGCGACACCCTCTACATCGCCACGGACGGCTCGGAGATGCCGGCCGAGATCGTGGAGCTGCACGAGATCGAGGTGGAGACCATCCCGGTGGAGACCATCGAGACCACAGTGGTGGGCGAGGAGGAGGAGGAGGACGACGACGACGAGGACGGCGGCGGTGGCGACCACGGCGGCGGGGGCGGCCACGGGCACGCCGGCCACCACCACCACCACCATCACCACCACCACCACCCGCCCATGATCGCTCTGCAGCCGCTGGTCACCGACGACCCGACCCAGGTGCACCACCACCAGGAGGTGATCCTGGTGCAGACGCGCGAGGAGGTGGTGGGCGGCGACGACTCGGACGGGCTGCGCGCCGAGGACGGCTTCGAGGATCAGATTCTCATCCCGGTGCCCGCGCCGGCCGGCGGCGACGACGACTACATTGAACAAACGCTGGTCACCGTGGCGGCGGCCGGCAAGAGCGGCGGCGGCGGCTCGTCGTCGTCGGGAGGCGGCCGCGTCAAGAAGGGCGGCGGCAAGAAGAGCGGCAAGAAGAGTTACCTCAGCGGCGGGGCCGGCGCGGCGGGCGGCGGCGGCGCCGACCCGGGCAACAAGAAGTGGGAGCAGAAGCAGGTGCAGATCAAGACCCTGGAGGGCGAGTTCTCGGTCACCATGTGGTCCTCAGATGAAAAAAAAGATATTGACCATGAGACAGTGGTTGAAGAACAGATCATTGGAGAGAACTCACCTCCTGATTATTCAGAATATATGACAGGAAAGAAACTTCCTCCTGGAGGAATACCTGGCATTGACCTCTCAGATCCCAAACAACTGGCAGAATTTGCTAGAATGAAGCCAAGAAAAATTAAAGAAGATGATGCTCCAAGAACAATAGCTTGCCCTCATAAAGGCTGCACAAAGATGTTCAGGGATAACTCGGCCATGAGAAAACATCTGCACACCCACGGTCCCAGAGTCCACGTCTGTGCAGAATGTGGCAAAGCTTTTGTTGAGAGTTCAAAACTAAAACGACACCAACTGGTTCATACTGGAGAGAAGCCCTTTCAGTGCACGTTCGAAGGCTGTGGGAAACGCTTTTCACTGGACTTCAATTTGCGCACACATGTGCGAATCCATACCGGAGACAGGCCCTATGTGTGCCCCTTCGATGGTTGTAATAAGAAGTTTGCTCAGTCAACTAACCTGAAATCTCACATCTTAACACATGCTAAGGCCAAAAACAACCAG**GGTATGGACTACAAGGATGACGATGACAAGGATTACAAAGACGACGATGATAAGGACTATAAGGATGATGACGACAAATGAGCTAGCACATAACTTACGGTAAATGGCCCGCCTGGCTGACCGCCCAACGACCCCCGCCCATTGACGTCAATAGTAACGCCAATAGGG |
| WNT9A plasmid |
| GCTAGCcaccatgctggatgggtccccgctggcgcgctggctggccgcggccttcgggctgacgctgctg  ctcgccgcgctgcgcccttcggccgcctacttcgggctgacgggcagcgagcccctgaccatcctcccgctga  ccctggagccagaggcggctgcccaggcgcactacaaggcctgcgaccggctgaagctggagcggaagc  agcggcgcatgtgccgccgggacccgggcgtggcagagacgctggtggaggccgtgagcatgagtgcg  ctcgagtgccagttccagttccgctttgagcgctggaactgcacgctggagggccgctaccgggccagcctgc  tcaagcgaggcttcaaggagactgccttcctctatgccatctcctcggctggcctgacgcacgcactggccaag  gcgtgcagcgcgggccgcatggagcgctgtacctgcgatgaggcacccgacctggagaaccgtgaggcct  ggcagtgggggggctgcggagacaaccttaagtacagcagcaagttcgtcaaggaattcctgggcagacg  gtcaagcaaggatctgcgagcccgtgtggacttccacaacaacctcgtgggtgtgaaggtgatcaaggctgg  ggtggagaccacctgcaagtgccacggcgtgtcaggctcatgcacggtgcggacctgctggcggcagttgg  cgcctttccatgaggtgggcaagcatctgaagcacaagtatgagacggcactcaaggtgggcagcaccacc  aatgaagctgccggcgaggcaggtgccatctccccaccacggggccgtgcctcgggggcaggtggcagc  gacccgctgccccgcactccagagctggtgcacctggatgactcgcctagcttctgcctggctggccgcttctcc  ccgggcaccgctggccgtaggtgccaccgtgagaagaactgcgagagcatctgctgtggccgcggccata  acacacagagccgggtggtgacaaggccctgccagtgccaggtgcgttggtgctgctatgtggagtgcagg  cagtgcacgcagcgtgaggaggtctacacctgcaagggctgaGCGGCCGC |
| FZD8 plasmid |
| GGATCCgccaccatggagtggggttacctgttggaagtgacctcgctgctggccgccttggcgctgctgca  gcgctctagcggcgctgcggccgcctcggccaaggagctggcatgccaagagatcaccgtgccgctgtgta  agggcatcggctacaactacacctacatgcccaatcagttcaaccacgacacgcaagacgaggcgggcctg  gaggtgcaccagttctggccgctggtggagatccagtgctcgcccgatctcaagttcttcctgtgcagcatgtac  acgcccatctgcctagaggactacaagaagccgctgccgccctgccgctcggtgtgcgagcgcgccaaggc  cggctgcgcgccgctcatgcgccagtacggcttcgcctggcccgaccgcatgcgctgcgaccggctgcccga  gcaaggcaaccctgacacgctgtgcatggactacaaccgcaccgacctaaccaccgccgcgcccagcccgc  cgcgccgcctgccgccgccgccgcccggcgagcagccgccttcgggcagcggccacggccgcccgccgg  gggccaggcccccgcaccgcggcggcggcaggggcggtggcggcggggacgcggcggcgcccccag  ctcgcggcggcggcggtggcgggaaggcgcggccccctggcggcggcgcggctccctgcgagcccggg  tgccagtgccgcgcgcctatggtgagcgtgtccagcgagcgccacccgctctacaaccgcgtcaagacagg  ccagatcgctaactgcgcgctgccctgccacaacccctttttcagccaggacgagcgcgccttcaccgtcttctg  gatcggcctgtggtcggtgctctgcttcgtgtccaccttcgccaccgtctccaccttccttatcgacatggagcgct  tcaagtacccggagcggcccattatcttcctctcggcctgctacctcttcgtgtcggtgggctacctagtgcgcct  ggtggcgggccacgagaaggtggcgtgcagcggtggcgcgccgggcgcggggggcgctgggggcgc  gggcggcgcggcggcgggcgcgggcgcggcgggcgcgggcgcgggcggcccgggcgggcgcggc  gagtacgaggagctgggcgcggtggagcagcacgtgcgctacgagaccaccggccccgcgctgtgcacc  gtggtcttcttgctggtctacttcttcggcatggccagctccatctggtgggtgatcttgtcgctcacatggttcctg  gcggccggtatgaagtggggcaacgaagccatcgccggctactcgcagtacttccacctggccgcgtggctt  gtgcccagcgtcaagtccatcgcggtgctggcgctcagctcggtggacggcgacccggtggcgggcatctg  ctacgtgggcaaccagagcctggacaacctgcgcggcttcgtgctggcgccgctggtcatctacctcttcatcg  gcaccatgttcctgctggccggcttcgtgtccctcttccgcatccgctcggtcatcaagcaacaggacggcccca  ccaagacgcacaagctggagaagctgatgatccgcctgggcctgttcaccgtgctctacaccgtgcccgccg  cggtggtggtcgcctgcctcttctacgagcagcacaaccgcccgcgctgggaggccacgcacaactgcccgt  gcctgcgggacctgcagcccgaccaggcacgcaggcccgactacgccgtcttcatgctcaagtacttcatgtg  cctagtggtgggcatcacctcgggcgtgtgggtctggtccggcaagacgctggagtcctggcgctccctgtgc  acccgctgctgctgggccagcaagggcgccgcggtgggcgggggcgcgggcgccacggccgcggggg  gtggcggcgggccggggggcggcggcggcgggggacccggcggcggcggggggccgggcggcgg  cgggggctccctctacagcgacgtcagcactggcctgacgtggcggtcgggcacggcgagctccgtgtctta  tccaaagcagatgccattgtcccaggtcCTCGAG |

Sg-YY1


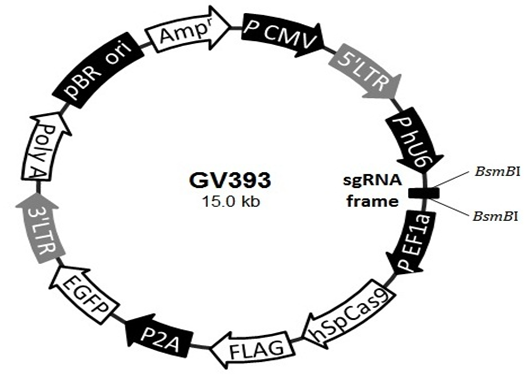


OE-YY1


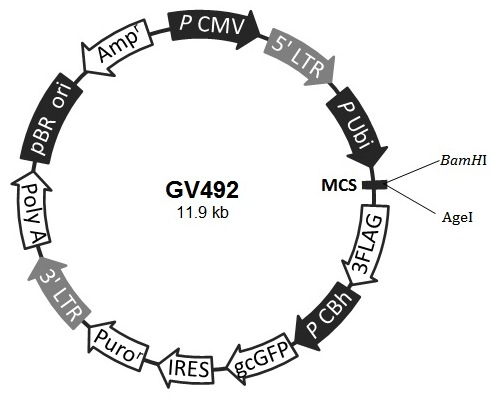


FZD8

WNT9A
