# Supplementary material for: Yin Yang 1 promotes the neuroendocrine differentiation of prostate cancer cells via the non‐canonical WNT pathway (FYN/STAT3)
Source: Clin Transl Med. 2023 Sep 28;13(10):e1422. doi: 10.1002/ctm2.1422 (PMC10539684; doi:10.1002/ctm2.1422)
Supplement: Supplementary file 5 — Table S4. The information of antibodies. [file CTM2-13-e1422-s004.docx]

Supplemental Table 4. The information of antibodies.

| Antibodies | Source | Identifier | application |
| --- | --- | --- | --- |
| YY1 | Cell signaling technology | 46395 | WB, IP, CHIP, IHC |
| FZD8 | Affinity | DF4931 | WB, IF |
| STAT3 | Abcam | ab68153 | WB |
| p-STAT3 | Abcam | ab267373 | WB |
| FYN | Abcam | ab184276 | WB, IP |
| p-FYN | Abcam | ab188319 | WB |
| β-catenin | Proteintech | 51067-2-AP | WB |
| WNT9A | Abcam | ab125957 | IF |
| Vimentin | Proteintech | 10366-1-AP | IF, WB |
| E-cadherin | Proteintech | 20874-1-AP | WB |
| N-cadherin | Proteintech | 22018-1-AP | IF, WB, IHC |
| SYP | Proteintech | 17785-1-AP | WB, IHC |
| NSE | Proteintech | 10149-1-AP | WB |
| CHgA | Proteintech | 10529-1-AP | WB |
| β-actin | Proteintech | 66009-1-Ig | WB |
| Ubiquitin | Cell signaling technology | 3936 | WB |
| Ki-67 | Beyotime | AF1738 | IF |
| CD56 | Cell signaling technology | 99746 | IF |
| Smurf2 | Abcam | ab53316 | WB |
| DVL | SANTA CRUZ BIOTECHNOLOGY | SC166303 | WB, IP |
