# Supplementary material for: Yin Yang 1 promotes the neuroendocrine differentiation of prostate cancer cells via the non‐canonical WNT pathway (FYN/STAT3)
Source: Clin Transl Med. 2023 Sep 28;13(10):e1422. doi: 10.1002/ctm2.1422 (PMC10539684; doi:10.1002/ctm2.1422)
Supplement: Supplementary file 6 — Table S5. Information on the predicted binding site and motifs of YY1 to the FZD8 promoter. [file CTM2-13-e1422-s003.docx]

Supplemental Table 5. Information on the predicted binding site and motifs of YY1 to the FZD8 promoter.

| Matrix ID | Name | Score | Relative score | Sequence ID | Start | End | Strand | Predicted sequence |
| --- | --- | --- | --- | --- | --- | --- | --- | --- |
| MA0095.1 | MA0095.1.YY1 | 8.191275 | 0.990201 | NC_000010.11:c35638246-35636247 | 1884 | 1889 | - | ACCATC |
| MA0095.1 | MA0095.1.YY1 | 8.191275 | 0.990201 | NC_000010.11:c35638246-35636247 | 1958 | 1963 | - | ACCATC |
| MA0095.1 | MA0095.1.YY1 | 7.388649 | 0.949208 | NC_000010.11:c35638246-35636247 | 1602 | 1607 | - | TCCATC |
| MA0095.1 | MA0095.1.YY1 | 7.219338 | 0.940561 | NC_000010.11:c35638246-35636247 | 1528 | 1533 | + | GCCATA |
| MA0095.1 | MA0095.1.YY1 | 7.027483 | 0.930762 | NC_000010.11:c35638246-35636247 | 86 | 91 | + | ACCATA |
| MA0095.1 | MA0095.1.YY1 | 6.399622 | 0.898695 | NC_000010.11:c35638246-35636247 | 1114 | 1119 | - | CCCATC |
| MA0095.1 | MA0095.1.YY1 | 6.399622 | 0.898695 | NC_000010.11:c35638246-35636247 | 1136 | 1141 | - | CCCATC |
| MA0095.1 | MA0095.1.YY1 | 6.224856 | 0.889769 | NC_000010.11:c35638246-35636247 | 149 | 154 | - | TCCATT |
| MA0095.1 | MA0095.1.YY1 | 6.224856 | 0.889769 | NC_000010.11:c35638246-35636247 | 529 | 534 | + | TCCATT |
| MA0095.1 | MA0095.1.YY1 | 6.224856 | 0.889769 | NC_000010.11:c35638246-35636247 | 1288 | 1293 | + | TCCATA |
